# OX40 controls effector CD4^+^ T-cell expansion, not follicular T helper cell generation in acute *Listeria* infection

**DOI:** 10.1002/eji.201344211

**Published:** 2014-05-21

**Authors:** Clare L Marriott, Emma C Mackley, Cristina Ferreira, Marc Veldhoen, Hideo Yagita, David R Withers

**Affiliations:** 1 MRC Centre for Immune Regulation, Institute for Biomedical Research, College of Medical and Dental Sciences, University of BirminghamBirmingham, United Kingdom; 2 Babraham Institute, Babraham Research CampusCambridge, United Kingdom; 3 Department of Immunology, Juntendo University School of MedicineTokyo, Japan

**Keywords:** CD4^+^ T cells, Costimulation, Germinal centre, Memory, OX40

## Abstract

To investigate the importance of OX40 signals for physiological CD4^+^ T-cell responses, an endogenous antigen-specific population of CD4^+^ T cells that recognise the 2W1S peptide was assessed and temporal control of OX40 signals was achieved using blocking or agonistic antibodies (Abs) in vivo. Following infection with *Listeria monocytogenes* expressing 2W1S peptide, OX40 was briefly expressed by the responding 2W1S-specific CD4^+^ T cells, but only on a subset that co-expressed effector cell markers. This population was specifically expanded by Ab-ligation of OX40 during priming, which also caused skewing of the memory response towards effector memory cells. Strikingly, this greatly enhanced effector response was accompanied by the loss of T follicular helper (TFH) cells and germinal centres. Mice deficient in OX40 and CD30 showed normal generation of TFH cells but impaired numbers of 2W1S-specific effector cells. OX40 was not expressed by 2W1S-specific memory cells, although it was rapidly up-regulated upon challenge whereupon Ab-ligation of OX40 specifically affected the effector subset. In summary, these data indicate that for CD4^+^ T cells, OX40 signals are important for generation of effector T cells rather than TFH cells in this response to acute bacterial infection.

## Introduction

Secondary lymphoid tissue supports the adaptive immune responses that provide effector cells to clear danger whilst also generating memory cells that establish protection for the future. Whilst peptide:MHC complexes and B7 family member costimulation are critical for productive T-cell responses, members of the TNF receptor superfamily are also involved. Amongst the TNF receptor superfamily, OX40 is a key determinant for the success of T-cell responses 1,2. Expression of OX40 is absent on naive T cells, but rapidly expressed upon activation although reportedly only maintained for a further 3–4 days 1–3. Signalling through OX40 was found to inhibit apoptotic pathways through expression of survivin 4 and Bcl-2 family members, tipping the balance in favour of enhanced survival and thus aiding clonal expansion 5. Consistent with this, OX40^−/−^ mice show impaired CD4^+^ T-cell responses, with reduced numbers of antigen-specific CD4^+^ T cells late in the primary response and the memory cell compartment is greatly reduced 6,7. Mice deficient in both OX40 and CD30 show even more impaired CD4^+^ T-cell responses and loss of memory cells, indicating redundancy due to shared signalling pathways 8,9. Provision of agonistic anti-OX40 antibodies (Abs) during a primary response resulted in greatly expanded numbers of antigen-specific CD4^+^ T cells and a larger memory cell compartment 6.

Expression of OX40 has been reported on memory phenotype (CD44^hi^CD62L^−^) CD4^+^ T cells within the spleen as well as on memory TCR transgenic cells 8, suggesting that OX40 signalling may also be important for memory cell survival. Provision of OX40L by ROR-γ^+^ innate lymphoid cells (ILCs), which constitutively express this molecule, has been proposed to explain the loss of memory CD4^+^ T cells in mice lacking these cells 10. T follicular helper (TFH) cells have also been described as OX40^+^ 11, at least in some situations 12 and OX40 expression is thought to be linked to their generation 11. Treg cells constitutively express OX40 although the functional consequences of this in vivo are not completely clear. In studies with OX40^−/−^ Treg cells, it is difficult to dissect impaired survival with impaired suppressive function in vivo 13,14.

Whilst a wealth of experiments on the importance of OX40 for T-cell responses have been performed, antigen-specific responses have used monoclonal TCR transgenic T cells 5,8, transferred in numbers thousands of times greater than a natural antigen-specific naive T-cell pool, a methodology that can alter the survival kinetics 15. In this study, we have analysed the response of an endogenous polyclonal population, with the aim to test in vivo when exactly OX40 was expressed, by what T-cell subsets and what the in vivo consequences of ligating or blocking this receptor were. We show that ligation of OX40, which is expressed principally by effector CD4^+^ T cells during priming, dramatically skewed the CD4^+^ T-cell response towards the formation of effector cells. Crucially, this occurred at the expense of TFH cells, resulting in a loss of germinal centres (GCs). Combined, these data clarify the role of OX40 signals for a physiological cohort of antigen-specific CD4^+^ T cells during an acute bacterial infection.

## Results

### Antigen-specific memory CD4^+^ T-cell survival is not impaired by blocking OX40L Abs

**Figure 1:**
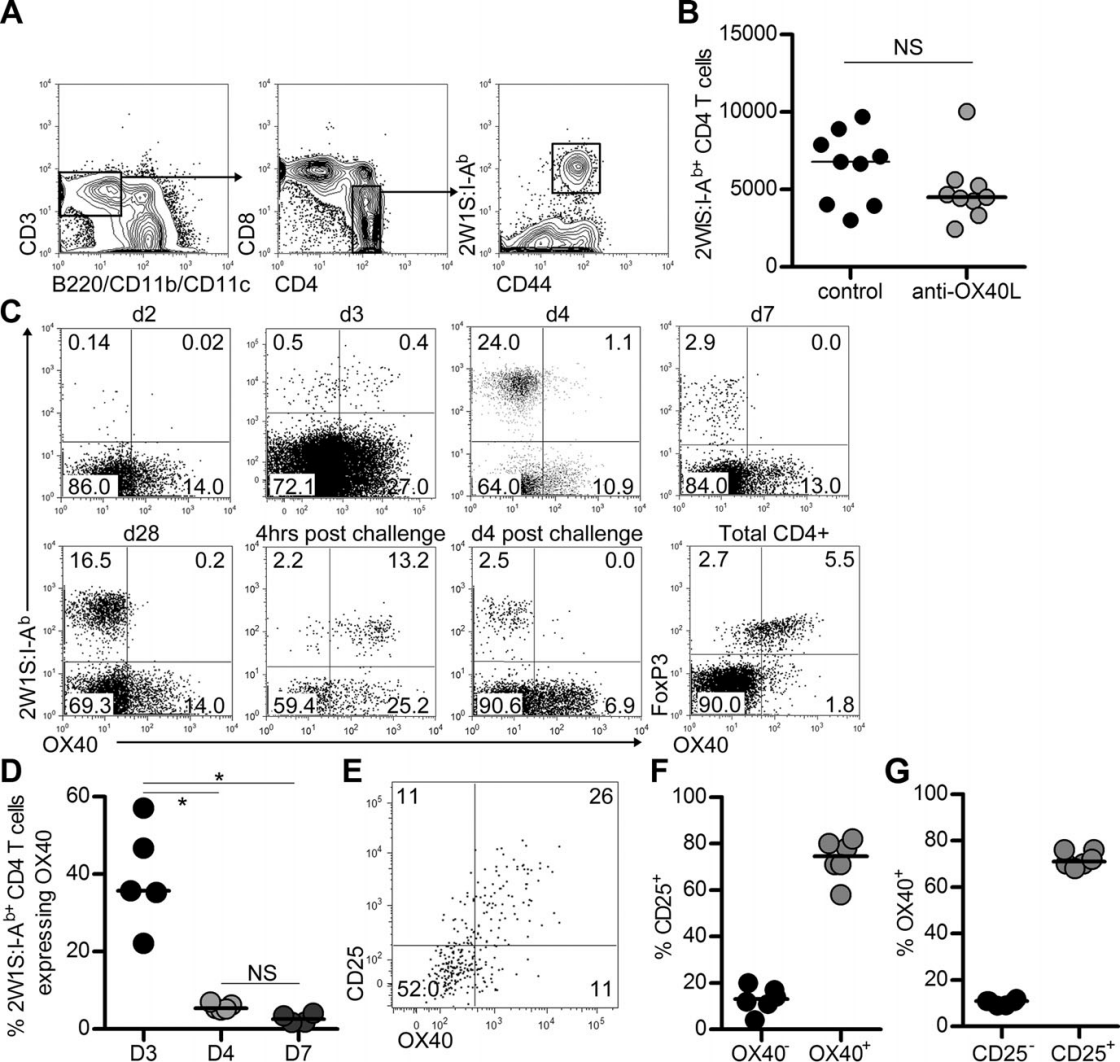
Blockade of OX40:OX40L interactions does not affect memory CD4^+^ T-cell survival. WT mice were immunised with Lm-2W and after 4 weeks given blocking anti-OX40L or control Abs twice weekly for 4 weeks. (A) Detection of CD44^hi^2W1S:I-A^b+^CD4^+^ T cells. Plots are gated on CD3^+^ B220^−^CD11b^−^CD11c^−^ followed by CD4^+^CD8^−^_,_ CD44^hi^2W1S:I-A^b+^ T cells. (B) Enumeration of CD44^hi^2W1S:I-A^b+^ CD4^+^ memory T cells in mice receiving either anti-OX40L or control IgG Abs. (C) Expression of OX40 on 2W1S:I-A^b+^CD4^+^ T cells at d2, d3, d4, d7 and d28 post-immunisation with Lm-2W, 4 hours and 4 days post-secondary challenge, and on Foxp3^+^CD4^+^ Treg cells. (D) Percentage of CD44^hi^ 2W1S:I-A^b+^ CD4^+^ T cells expressing OX40 at d3, d4 and d7 post-immunisation. (E) Expression of CD25 and OX40 on 2W1S:I-A^b+^ CD4^+^ T cells at 3 dpi. (F) Percentage of OX40^−^ and OX40^+^CD44^hi^2W1S:I-A^b+^CD4^+^ T cells that express CD25. (G) Percentage of CD25^−^ and CD25^+^CD44^hi^2W1S:I-A^b+^CD4^+^ T cells that express OX40. (A, C) Plots are representative of ≥6 mice pooled from two independent experiments. (B, D, F, G) Data are pooled from two independent experiments, each data point represents one mouse. Bars show medians. Mann–Whitney test, *p < 0.05, NS = non-significant.

Signals through OX40 are clearly important for the survival of CD4^+^ T cells after priming 5, however, whether they are important for the continued survival of formed memory cells is controversial 2. We sought to investigate the importance of OX40 signals for the survival of an endogenous antigen-specific memory cell pool, using the well-characterised response to an attenuated *Listeria monocytogenes* strain expressing the 2W1S peptide (Lm-2W) 16. In this response, the memory phase occurs from 3–4 weeks post-infection, after rapid clearance of the bacteria. Therefore, WT mice were immunised with Lm-2W and after 4 weeks given twice weekly injections of anti-OX40L (or control) Abs for a further 28 days. At this point, numbers of CD44^hi^ 2W1S:I-A^b+^ CD4^+^ T cells were enumerated (Fig.1A). Whilst there was a modest reduction in the number of CD44^hi^2W1S:I-A^b+^CD4^+^ T cells recovered from the control and treated mice, this difference was not significant (Fig.1B; WT vs. OX40L: *p* = 0.2973; median for control: 6794, anti-OX40L: 4509).

### Heterogeneous expression of OX40 by 2W1S:I-A^b+^ CD4^+^ T cells

Given that the survival of 2W1S-specific memory T cells was not significantly impaired by anti-OX40L Abs, expression of OX40 by 2W1S-specific CD4^+^ T cells during the response to Lm-2W infection was assessed, with total CD4^+^ Treg cells used as a positive control for OX40 detection (Fig.1C). Although only a small number of 2W1S:I-A^b+^CD4^+^ T cells were detectable 2 days post-infection (dpi) with Lm-2W, these lacked expression of OX40 (Fig.1C). By 3 dpi, OX40 expression was detected on the 2W1S:I-A^b+^ CD4^+^ T cells, however <50% of the cells were OX40^+^ (Fig.1C and D) and this represented the peak of detectable OX40 expression since by 4 dpi approximately 5% of CD44^hi^2W1S:I-A^b+^CD4^+^ T cells expressed this receptor. These data were notably different to that described for TCR transgenic T cells, where OX40 was expressed by all the antigen-specific cells 5,17,18. Following Lm-2W infection, three subsets of 2W1S-specific CD4^+^ T cells have been elegantly described 19: CXCR5^−^PD-1^−^T-bet^+^ effector T cells (where PD-1 is programmed death-1), CXCR5^+^PD-1^−^Bcl-6^+^ cells that give rise to central memory cells and CXCR5^+^PD-1^+^Bcl-6^+^ TFH cells. Expression of CD25 can be used at 3 dpi to identify the CXCR5^−^PD-1^−^T-bet^+^ effector T-cell subset 20. Strikingly, the majority (>70%) of CD25^+^ 2W1S-specific T cells at 3 dpi expressed OX40 and accounted for the majority (>70%) of the OX40-expressing CD44^hi^2W1S:I-A^b+^CD4^+^ T cells (Fig.1E–G). By 7 dpi, no OX40 expression was detected on CD44^hi^2W1S:I-A^b+^ CD4^+^ T cells (Fig.1C and D), including the TFH population. Since OX40 signals have been implicated in TFH formation and survival 8, we investigated whether OX40^+^ cells co-expressed markers of TFH cells. Expression of Bcl-6 was detected from 4 dpi and although only a fraction of the CD44^hi^2W1S:I-A^b+^CD4^+^ T cells expressed OX40 at this time, a minority of the cells co-expressed Bcl-6 (Supporting Information Fig. 1). Therefore, whilst OX40 is expressed mostly by 2W1S-specific CD4^+^ T cells with an effector phenotype, a subset of Bcl-6-expressing 2W1S-specific CD4^+^ T cells do also express OX40.

To further investigate whether OX40 signals were required for the formation of TFH cells in the response to Lm-2W, mice deficient in both OX40 and CD30 were used, since there may be redundancy in these signalling pathways 8. WT and CD30^−/−^OX40^−/−^ mice were immunised with Lm-2W and then analysed at 7 dpi. An overall decrease in the number of CD44^hi^2W1S:I-A^b+^CD4^+^ T cells (Supporting Information Fig. 2; *p* = 0.0317; median for WT: 52 331, CD30^−/−^OX40^−/−^: 21 975) resulted from less CXCR5^−^ effector cells (*p* = 0.0159; median for WT: 27 164, CD30^−/−^OX40^−/−^: 8103) consistent with decreased survival, however generation of TFH was not impaired (*p* = 0.5556; median for WT: 2850, CD30^−/−^OX40^−/−^: 3430), indicating that the generation of these cells did not require OX40 signals.

Although memory phenotype CD4^+^ T cells have been shown to express OX40, amongst the memory CD44^hi^2W1S:I-A^b+^CD4^+^ T-cell population no OX40 expression was detected. The unimpaired survival of these cells when OX40L signals were blocked is likely explained by a lack of OX40 expression by the antigen-specific CD4^+^ T cells at this time. These cells could rapidly up-regulate OX40 expression upon challenge in vivo with 2W1S peptide (Fig.1C) as previously described 2. Here, the vast majority of 2W1S:I-A^b+^CD4^+^ T cells expressed OX40, indicating that expression at this stage was not restricted to the effector subset. Similarly, if Lm-2W-infected mice were challenged at 7 dpi with 2W1S peptide, OX40 was rapidly expressed by almost all 2W-specific CD4^+^ T cells (Supporting Information Fig. 3). These data show that OX40 expression by an endogenous polyclonal antigen-specific population of CD4^+^ T cells is very tightly regulated temporally and not uniformly expressed amongst responding cells in a primary response. Upon re-encounter of antigen, OX40 is more broadly expressed.

### Agonistic anti-OX40 Abs at challenge drive effector T-cell expansion

**Figure 2:**
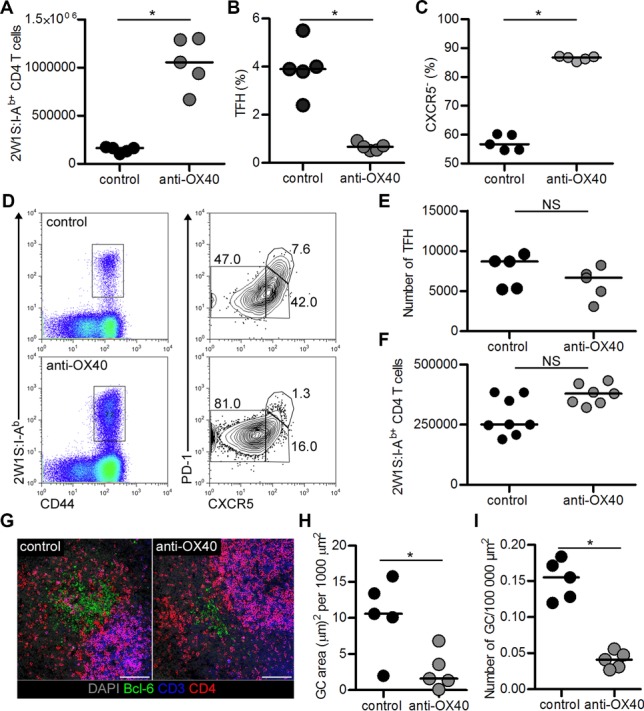
Ligation of OX40 upon challenge enhances memory cell expansion and skews phenotype towards T effector memory. Secondary lymphoid tissues were analysed from mice receiving agonistic anti-OX40 or control IgG Abs after challenge. (A) Enumeration of CD44^hi^2W1S:I-A^b+^CD4^+^ T cells given Abs on the day of challenge. Percentage of CD44^hi^2W1S:I-A^b+^CD4^+^ T cells that are (B) CXCR5^+^PD-1^+^ TFH cells or (C) CXCR5^−^PD-1^−^ effector cells. (D) Expression pattern of CXCR5 and PD-1 by CD44^hi^2W1S:I-A^b+^CD4^+^ T cells. (E) Total number of CD44^hi^2W1S:I-A^b+^CD4^+^ CXCR5^+^ PD-1^+^ TFH cells. (F) Enumeration of CD44^hi^2W1S:I-A^b+^ CD4^+^ T cells from mice given Abs the day after challenge. (G) Immunofluorescence staining of spleen sections DAPI (grey) and expression of Bcl-6 (green), CD3 (blue) and CD4 (red) shown. Magnification 25×, scale bar represents 100 μm. Confocal images are representative of five mice. (H) Area of Bcl-6^+^ germinal centre (GC) per 1000 μm^2^ spleen. (I) Absolute number of GCs per 100 000 μm^2^ spleen. (A–C, E, H, I) Data are pooled from two independent experiments, each data point represents one mouse. Bars show medians. (D) Plots are representative of ≥6 mice pooled from two independent experiments. Mann–Whitney test, *p < 0.05, NS = non-significant.

Although the survival of 2W1S-specific cells in the memory phase of the response was OX40 independent, the rapid expression of OX40 within hours of seeing cognate antigen again prompted us to test the effect of OX40 ligation at this time. Provision of agonistic anti-OX40 Abs 6 on the day of challenge resulted in significantly more (approximately 6.4-fold) CD44^hi^2W1S:I-A^b+^CD4^+^ T cells 4 days later (Fig.2A; *p* = 0.0079; median for control: 164 070, anti-OX40: 1 055 000). The majority of these 2W1S:I-A^b+^CD4^+^ T cells were CXCR5^−^PD-1^−^ effector CD4^+^ T cells 19 and the percentage of CXCR5^+^PD-1^+^ TFH-cell population significantly reduced (Fig.2B–D). However, total numbers of TFH cells were comparable, indicating that OX40 ligation had specifically expanded effector 2W1S-specific cells (Fig.2E; *p* = 0.222; median for control: 8712, anti-OX40: 6673). If provision of anti-OX40 Abs was delayed until the day after challenge, no significant effect on the number of 2W1S:I-A^b+^CD4^+^ T cells was detected, indicating that as with a primary response, expression of OX40 was rapidly lost by the 2W1S:I-A^b+^CD4^+^ T cells (Fig.2F; *p* = 0.0541; median for control: 250 936, anti-OX40: 379 040). To investigate whether the reduced percentage of TFH cells impacted the ability to sustain GCs, sections of spleen at day 4 post-challenge were stained for Bcl-6, the key transcription factor expressed by GC B cells and TFH cells. Despite no difference in absolute number of TFH cells, spleens from anti-OX40-treated mice were considerably larger than that of control mice resulting in less TFH cells per unit area. In mice given anti-OX40 Abs, fewer GCs per unit area of spleen were detected, suggesting that changes to the balance of the T-cell response had impacted the ability to sustain these structures (Fig.2G–I).

### Ligation of OX40 drives effector T-cell formation at the expense of GCs in a primary response

**Figure 3:**
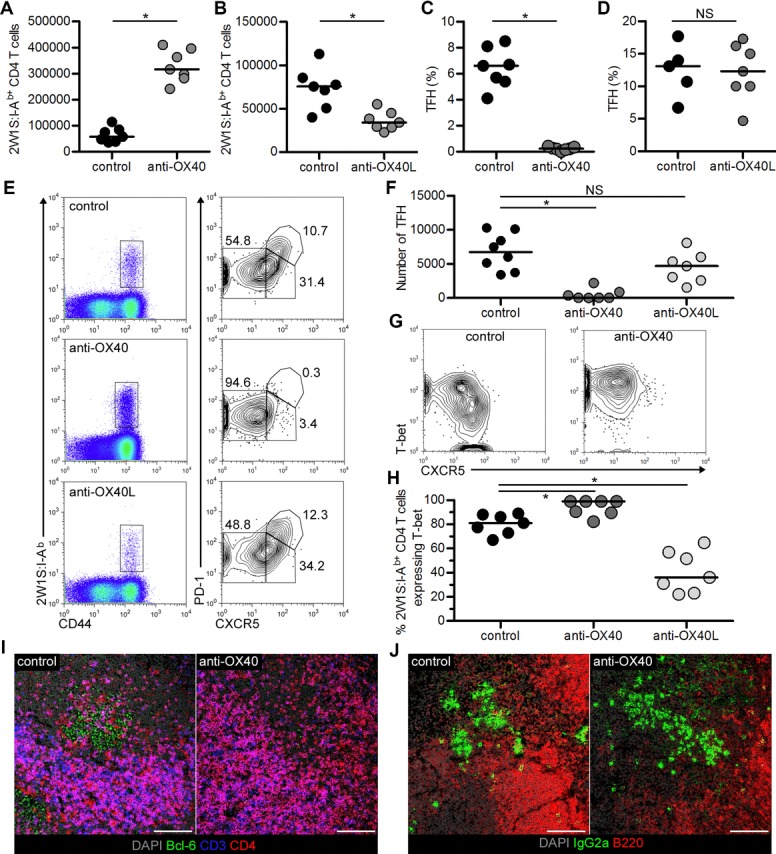
Therapeutic manipulation of OX40:OX40L interactions impact on the size and phenotype of the primary 2W1S:I-A^b+^CD4^+^ T-cell response. Analysis of CD44^hi^2W1S:I-A^b+^CD4^+^ T cells at 7 dpi from mice given anti-OX40, anti-OX40L or control IgG Abs. (A) Enumeration of CD44^hi^2W1S:I-A^b+^ CD4^+^ T cells in spleen at 7 dpi in mice given anti-OX40 Abs; or (B) anti-OX40L Abs. (C) Percentage of CD44^hi^2W1S:I-A^b+^CD4^+^ cells that are CXCR5^+^PD-1^+^ TFH cells following treatment with anti-OX40 or (D) anti-OX40L Abs. (E) Representative CXCR5 and PD-1 expression on CD44^hi^2W1S:I-A^b+^CD4^+^ T cells in mice given control IgG, anti-OX40 or anti-OX40L. (F) Total numbers of CD44^hi^2W1S:I-A^b+^CD4^+^CXCR5^+^PD-1^+^ TFH cells from mice receiving control IgG, anti-OX40 or anti-OX40L Abs. (G) Representative CXCR5 and T-bet expression on CD44^hi^ 2W1S:I-A^b+^ CD4^+^ T cells in mice given control IgG or anti-OX40. (H) Percentage of CD44^hi^2W1S:I-A^b+^CD4^+^ T cells expressing T-bet in mice receiving control IgG, anti-OX40 Abs or anti-OX40L. (I) Immunofluorescence staining of spleen sections from 7 dpi, DAPI (grey) and expression of Bcl-6 (green), CD3 (blue) and CD4 (red) shown, and (J) DAPI (grey) and expression of IgG2a (green) and B220 (red) shown. Magnification 25×, scale bar represents 100 μm. Confocal images are representative of five mice (I) or two mice (J). (A–D, F and H) Data are pooled from two independent experiments, each data point represents one mouse. Bars show medians. (E, G) Plots are representative of ≥6 mice pooled from two independent experiments. Mann–Whitney test, *p < 0.05, NS = non-significant.

OX40 expression by 2W1S-specific cells was tightly restricted to a brief window of time associated with antigen exposure and proliferation. Since OX40 ligation at challenge caused the expansion of only the effector memory subset, we sought to investigate the effects of OX40 ligation during a primary response. Previously, agonistic anti-OX40 Abs have dramatically enhanced the numbers of TCR transgenic T cells 6,21. Mice were infected with Lm-2W, then given agonistic anti-OX40 Abs at 1 dpi and analysed at 7 dpi. Provision of anti-OX40 Abs resulted in approximately 5.5-fold more CD44^hi^2W1S:I-A^b+^CD4^+^ T cells (Fig.3A; *p* = 0.0006; median for controls: 57 710, anti-OX40: 316 688). In contrast, provision of the blocking anti-OX40L Abs at 1 and 3 dpi approximately halved the number of CD44^hi^2W1S:I-A^b+^CD4^+^ T cells at 7 dpi (Fig.3B; *p* = 0.0041; median for control: 75 684, anti-OX40L: 34 167). As detected at challenge, with anti-OX40, the CD44^hi^2W1S:I-A^b+^CD4^+^ T cells were heavily skewed towards CXCR5^−^ effector T cells. Furthermore, anti-OX40, but not anti-OX40L, Abs dramatically reduced the percentage of the 2W1S-specific cells with a TFH-cell phenotype (Fig.3C–E; *p* = 0.0006; median for control: 6.6%, anti-OX40: 0.24%). Analysis of total numbers of TFH cells confirmed these cells were almost absent in anti-OX40-treated mice (Fig.3F). Therefore, unlike at challenge where TFH cell numbers appeared undiminished, anti-OX40 Abs during priming result in the expansion of effector cells and the loss of the TFH-cell population.

Amongst CD44^hi^2W1S:I-A^b+^CD4^+^ T cells after exposure to Lm-2W, expression of T-bet is restricted to the CXCR5^−^ effector T cells 19 (Fig.3G). To further demonstrate that OX40 ligation caused the accumulation of the effector subset, we checked expression of T-bet amongst CD44^hi^2W1S:I-A^b+^CD4^+^ T cells. Ligation of OX40 resulted in the vast majority of 2W-specific CD4^+^ T cells expressing T-bet at 7 dpi (Fig.3G and H; control vs. anti-OX40: *p* = 0.0067; median for controls: 81%, anti-OX40: 99%; control vs. anti-OX40L: *p* = 0.0167; median for anti-OX40L: 56.9%). Finally, given the loss of the TFH-cell population, sections of spleen taken at 7 dpi were stained for expression of Bcl-6 to assess effects on GCs (Fig.3I). Whilst GCs were evident in control mice, in mice given agonistic anti-OX40 no clusters of Bcl-6^+^ cells were detected. The detection of IgG2a^+^ plasma cells indicated that a B-cell response with switching to Th1 Ab isotypes had been initiated (Fig.3J) consistent with previous data showing GCs are not required for initial Ab switching 22. These data show strong parallels to the recent study of anti-OX40 Abs in the response to lymphocytic choriomeningitis virus **(**LCMV). A key difference is that in this study, TCR transgenic T cells that homogenously express OX40 during priming were used. Ligation of OX40 caused up-regulation of Blimp-1 repressing TFH-cell formation. However, in the response to Lm-2W described here, expression of OX40 is restricted to cells with an effector phenotype, which then specifically benefit from Ab-ligation of the receptor. Whilst there is no previous evidence for the OX-86 clone depleting OX40^+^ cells, we assessed the numbers of Treg cells, which constitutively express OX40, in control and anti-OX40-treated mice. No significant differences were detected in the number of FoxP3^+^2W1S:I-A^b+^CD4^+^ T cells or total CD4^+^FoxP3^+^ T cells (Supporting Information Fig. 4), arguing against in vivo depletion of OX40^+^ cells by the anti-OX40 Abs.

These data indicated that Ab-mediated ligation of OX40 during priming caused the specific expansion of OX40^+^2W1S:I-A^b+^ CD4^+^ T cells, which gave rise to the expanded CXCR5^−^ effector T-cell pool. Consistent with this, administration of anti-OX40 Abs at 4 dpi with Lm-2W, a time point when the CD44^hi^2W1S:I-A^b+^ CD4^+^ T cells had lost expression of OX40, resulted in only a very modest increase in CD44^hi^2W1S:I-A^b+^ CD4^+^ T cells (Supporting Information Fig. 5; *p* = 0.0047; median for control: 46 097, agonistic anti-OX40: 59 423) and the percentage of TFH cells was not affected.

### Ligation of OX40 results in an enhanced effector memory T-cell pool

**Figure 4:**
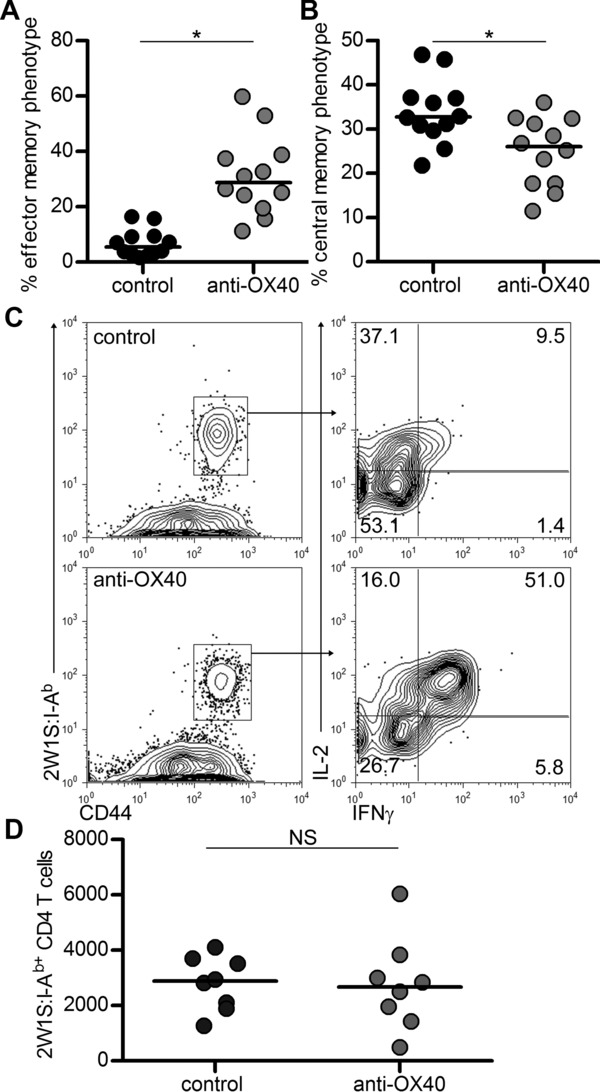
Ligation of OX40 alters the phenotype but not the number of 2W1S:I-A^b+^ memory CD4^+^ T cells. Analysis of the CD44^hi^2W1S:I-A^b+^ memory CD4^+^ T cells. The percentages of the memory CD4^+^ T-cell population with (A) effector memory (IL-2^+^IFN-γ^+^) or (B) central memory (IL-2^+^IFN-γ^−^) phenotype are shown. (C) Flow cytometry staining of cytokines IL-2 and IFN-γ following in vivo stimulation with 2W1S peptide at 28 dpi. (D) Total number of CD44^hi^2W1S:I-A^b+^ CD4^+^ memory T cells recovered after in vivo peptide stimulation. (A, B, D) Data have been pooled from ≥2 independent experiments, each data point represents one mouse. Bars show medians. (C) Plots are representative of ≥6 mice pooled from two independent experiments. Mann–Whitney test, *p < 0.05, NS = non-significant.

Given the increased effector T-cell expansion, anti-OX40 Abs likely enhanced the effector rather than central memory pool. Previous studies with OX40-deficient OTII T cells indicated that OX40 signals were important for effector rather than central memory generation 23. Recent data from a human OX40-deficient patient reported a reduced proportion of effector memory CD4^+^ T cells 24. To assess the memory population after OX40 ligation during priming, mice were immunised as before, treated with control or agonistic anti-OX40 Abs at 1 dpi and at 28 dpi given 2W1S peptide in vivo and analysed 4 hours later. In vivo peptide stimulation was used to functionally define effector memory cells as those able to make both IL-2 and IFN-γ, and central memory cells as those making only IL-2 20. In mice treated with anti-OX40 Abs, there was a significant increase in the percentage of CD44^hi^2W1S:I-A^b+^CD4^+^ T cells defined functionally as effector memory cells (Fig.4A and C). The enhanced frequency of effector memory cells was accompanied by a significant decrease in the central memory population (Fig.4B; *p* = 0.013), therefore ligation of OX40 during priming had significantly skewed the resulting memory population. Surprisingly, the total numbers of CD44^hi^2W1S:I-A^b+^CD4^+^ T cells between control and anti-OX40-treated mice, in the secondary lymphoid tissue at least, were now comparable, indicating a dramatic contraction of the primary response and normal homeostatic regulation of the memory pool size (Fig.4D).

### Intrinsic expression of T-bet is not required for effector T-cell expansion

**Figure 5:**
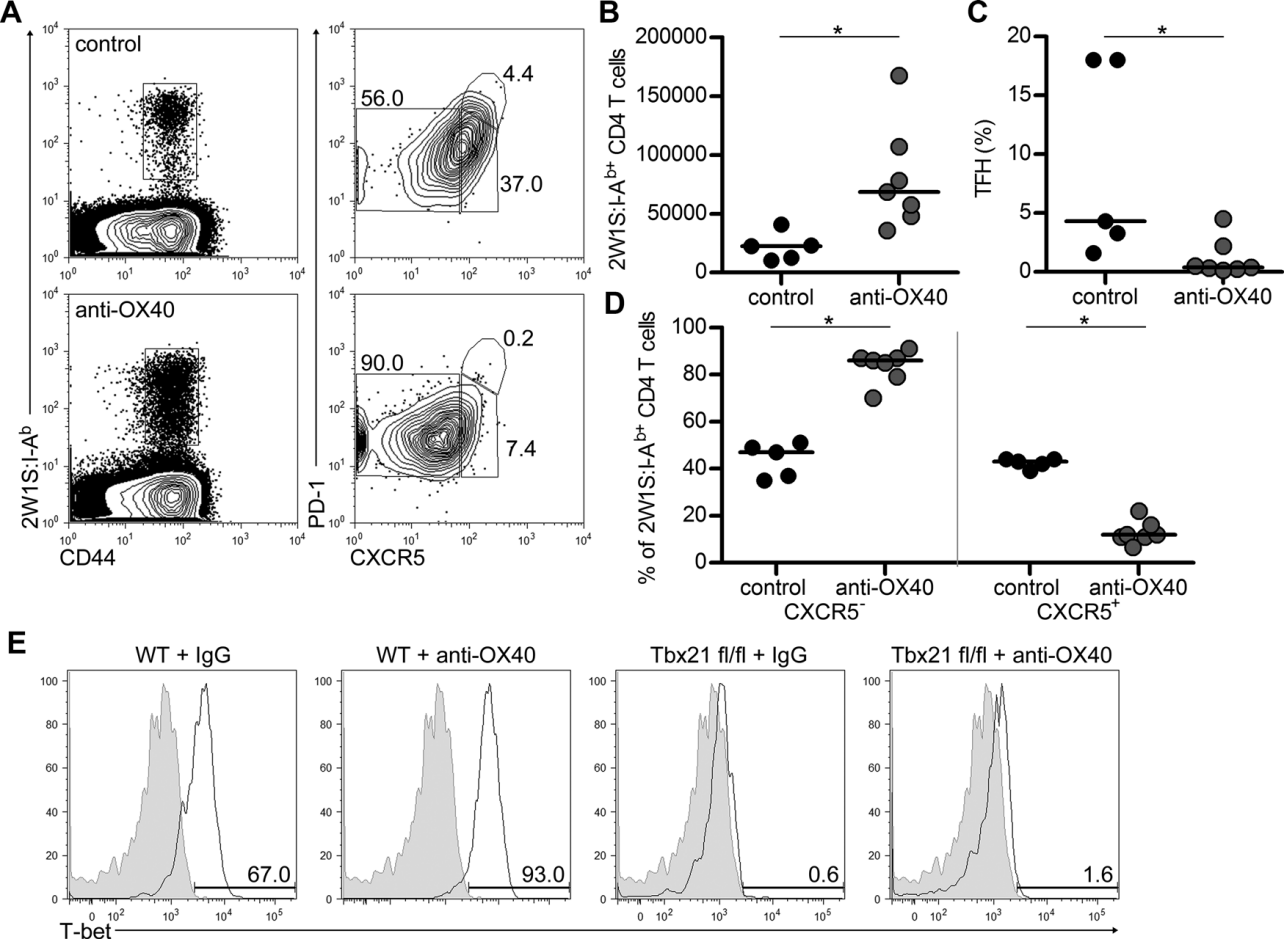
Intrinsic expression of T-bet is not required for the effects of agonistic anti-OX40 Abs. WT and Rag1-Cre Tbx21 fl/fl Rosa26-tdRFP were immunised with Lm-2W and given control IgG or anti-OX40 Abs at 1 dpi and then analysed at 7 dpi. (A) Staining of CD44^hi^2W1S:I-A^b+^CD4^+^ T cells for CXCR5 and PD-1 in Rag1-Cre Tbx21 fl/fl Rosa26-tdRFP. (B) Total numbers of CD44^hi^2W1S:I-A^b+^CD4^+^ T cells in Rag1-Cre Tbx21 fl/fl Rosa26-tdRFP mice. Percentage of (C) TFH cells and (D) CXCR5^−^ and CXCR5^+^ CD44^hi^2W1S:I-A^b+^CD4^+^ T cells from Rag1-Cre Tbx21 fl/fl Rosa26-tdRFP mice. (E) Expression of T-bet by CD44^hi^2W1S:I-A^b+^CD4^+^ T cells. (A, E) Plots are representative of ≥5 mice pooled from two independent experiments. (B–D) Data have been pooled from two independent experiments, each data point represents one mouse. Bars show medians. Mann–Whitney test, *p < 0.05, NS = non-significant.

Ligation of OX40 has been found to induce T-bet mRNA 21. Expression of T-bet is thought to block Th2 differentiation, rather than promote Th1 formation 25. To investigate whether the phenotypic changes observed with OX40 ligation were dependent upon endogenous T-bet expression, WT and Rag1-Cre Tbx21 fl/fl Rosa26-tdRFP were immunised with Lm-2W, given control or anti-OX40 Abs at 1 dpi and analysed at 7 dpi. In Rag1-Cre Tbx21 fl/fl Rosa26-tdRFP mice given control IgG, the response was comparable to WT mice (Fig.5A). Provision of anti-OX40 Abs resulted in significantly increased expansion of the CD44^hi^2W1S:I-A^b+^CD4^+^ T cells (Fig.5B; *p* = 0.005; median for control: 22 405, anti-OX40: 68 405) with again the loss of TFH cells (Fig.5C; *p* = 0.0344) and the skewing towards CXCR5^−^ cells (Fig.5D; CXCR5^−^, *p* = 0.005; CXCR5^+^, *p* = 0.005). Staining for expression of T-bet confirmed its absence in the Tbx21 fl/fl mice, demonstrating that the effects of anti-OX40 Abs on T-cell phenotype do not require T-bet expression, consistent with experiments with non-antigen-specific CD4^+^ T cells 21.

### Altered OX40 expression in a Th2 response, but still effector cell expansion upon ligation

**Figure 6:**
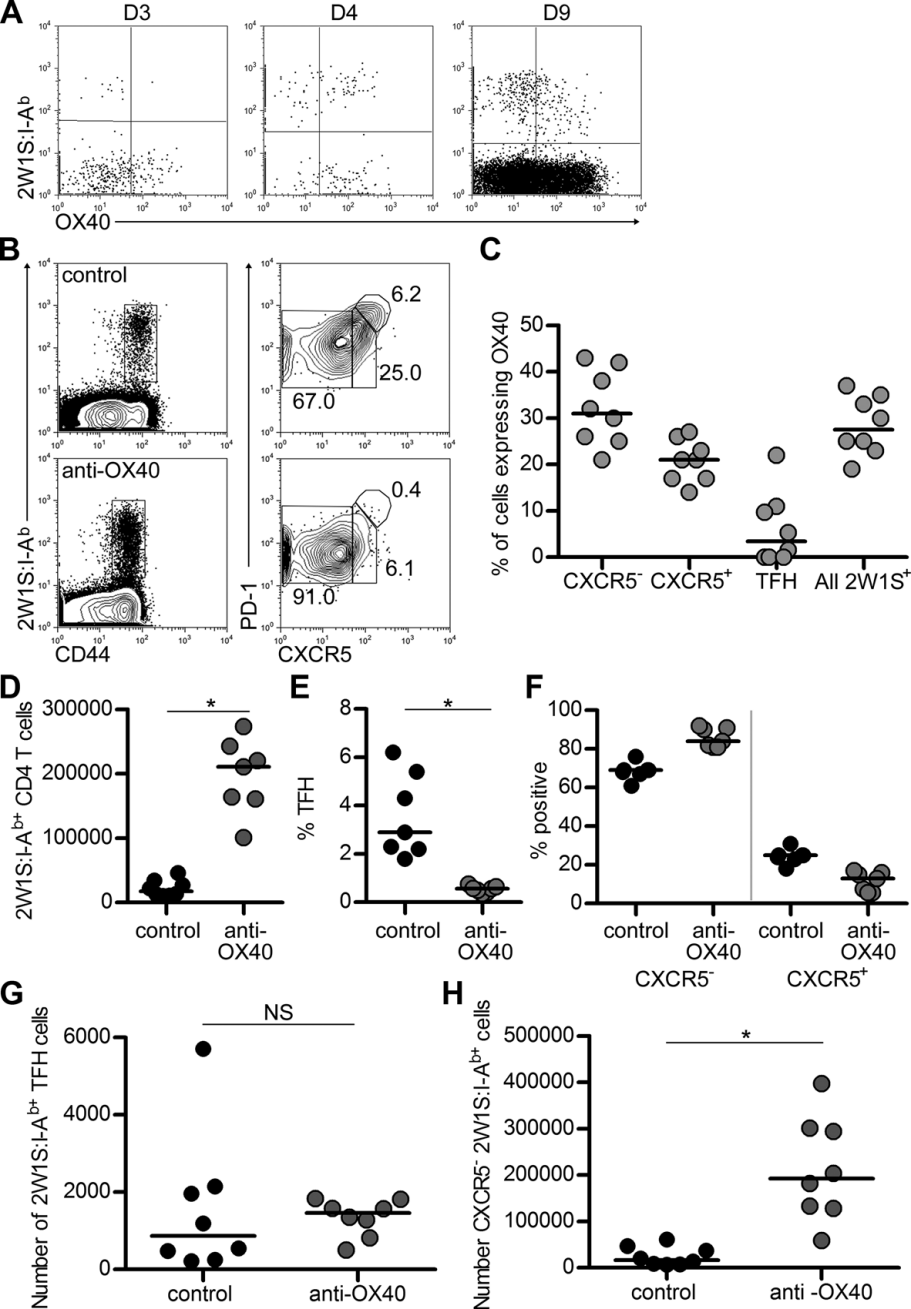
OX40 expression is prolonged with alum adjuvant, but ligation still causes effector cell expansion. Mice were immunised with alum-precipitated 2W1S peptide and given anti-OX40 Abs or control IgG at 1 dpi. (A) Representative plots of OX40 expression on CD44^hi^2W1S:I-A^b+^CD4^+^ T cells at d3, d4 and d9 post-immunisation. Plots are representative of ≥3 mice. (B) Staining of CD44^hi^2W1S:I-A^b+^CD4^+^ T cells for CXCR5 and PD-1 by flow cytometry at 9 dpi. (C) Percentage of CXCR5^−^, CXCR5^+^ and TFH cells amongst CD44^hi^2W1S:I-A^b+^CD4^+^ T cells, or all of CD44^hi^2W1S:I-A^b+^CD4^+^ T cells expressing OX40 at 9 dpi. (D) Enumeration of CD44^hi^2W1S:I-A^b+^CD4^+^ T cells at 9 dpi. (E) Percentage of TFH cells amongst CD44^hi^2W1S:I-A^b+^CD4^+^ T cells. (F) Percentage of CD44^hi^2W1S:I-A^b+^CD4^+^ T cells that are CXCR5^−^ or CXCR5^+^. Total number of CD44^hi^2W1S:I-A^b+^CD4^+^ T cells that are TFH cells (G) or CXCR5^−^ (H). (A and B) Plots are representative of ≥5 mice pooled from two independent experiments unless stated. (C–H) Data have been pooled from two independent experiments, each data point represents one mouse. Bars show medians. Mann–Whitney test, *p < 0.05, NS = non-significant.

To assess the response when 2W1S peptide was encountered within a different context, mice were immunised with 2W1S peptide precipitated with alum. This resulted in different kinetics of OX40 expression, with expression of OX40 by 2W1S-specific cells initiated at 4 dpi, and maintained until at least 9 dpi (Fig.6A). Furthermore, at 9 dpi, expression was not exclusively confined to CXCR5^−^ cells, although very little was detected on the TFH-cell population (Fig.6B and C). Ligation of OX40 had the same effect as in the Lm-2W infection, with the dramatic expansion of the 2W1S-specific CD4^+^ T cells (Fig.6B and D; *p* = 0.003; median for control: 17 721, anti-OX40: 210 626). Again, this expansion was specific to the CXCR5^−^ cells, which expanded significantly, whilst although the percentage of TFH cells was much reduced, absolute numbers were not significantly affected (Fig.6E–H) and the number of GC structures appeared comparable (data not shown). Therefore, whilst the manner in which the antigen is encountered changed the pattern of OX40 expression by responding cells, enhanced OX40 signalling again expanded specifically the effector population.

## Discussion

In this report, we show that for an endogenous polyclonal CD4^+^ T-cell population responding to *Listeria* infection, OX40 expression is induced during priming largely on responding antigen-specific T cells with an effector phenotype. Modulation of OX40 through blocking OX40L, ligation of OX40 or mice deficient in OX40, substantially affected the effector subset of 2W1S-specific CD4^+^ T cells. Although expression of OX40 has been previously linked to the persistence of GCs and TFH cells 8, within this infection model, the formation of TFH cells did not require OX40 signals and ligation of OX40 did not enhance their number.

Initially, we sought to analyse expression of OX40 by endogenous polyclonal CD4^+^ T cells, utilising MHC-II tetramers to build upon previous investigations reliant upon TCR transgenic T cells. In these studies, OX40 expression was induced within 24–48 hours and maintained for several days 1,5,6. Chronic LCMV infection will maintain antigen-specific CD4^+^ T-cell expression of OX40 for weeks in contrast to acute infection 17. Therefore, the kinetics of OX40 expression reflects the nature of the antigen and the manner in which it is encountered. The brief window of OX40 expression in the primary response to Lm-2W likely reflects the acute nature of this attenuated infection and its rapid clearance. Clearly, the other 2W1S-specific subsets can be induced to express OX40 upon re-encounter of the peptide. Immunisation with alum-precipitated 2W1S-peptide resulted in a longer window of OX40 expression by the same endogenous antigen-specific population. In all these situations, Ab-ligation of OX40 specifically enhanced the effector subset of 2W1S-specific cells. It was recently shown in LCMV infection that Ab-ligation of OX40 greatly enhanced the effector subset and caused the loss of TFH cells, an observation linked to enhanced Blimp-1 expression, thought to block Bcl-6 and TFH formation 18. In this interesting study, the authors propose that ligation of OX40 drives enhanced IL-2 expression, driving Blimp-1 expression and STAT5 activation and inhibiting TFH-cell formation. In the primary response to Lm-2W, the majority of the cells that express OX40 already appear committed to an effector phenotype, indicating that in this situation, OX40 ligation promotes the proliferation and survival of this effector population rather than impaired differentiation of the TFH cells. This expansion is also not dependent upon endogenous T-cell expression of T-bet. Impaired TFH-cell differentiation may well occur when OX40 is ligated upon re-exposure to antigen, where 2W1S-specific CD4^+^ T cells rapidly up-regulate OX40 regardless of their effector or TFH-cell phenotype. Notably, total numbers of TFH cells are not altered when OX40 is ligated at challenge, whilst in a primary response the effector cell expansion is accompanied by the complete loss of the TFH-cell population. These data suggest that during the primary response, the enhanced effector cell expansion is accompanied by the out-competition of the TFH cells, perhaps due to a loss of niches within the tissue. Alternatively, the high levels of IFN-γ produced following Ab-ligation of OX40 may cause the loss of TFH cells and GCs, analogous to CD70 transgenic mice 26.

Previous studies have concluded that signals through OX40 are important for persistence of TFH cells and thus the formation of T-dependent humoral responses and GCs 8. Boettler et al. have also shown that control of chronic LCMV through high-affinity Ab is dependent upon OX40 17. Therefore, it might have been anticipated that provision of anti-OX40 Abs would enhance the generation of TFH cells, however this clearly does not occur in our hands or others 18. The injection of large quantities of anti-OX40 Abs may deregulate the normal in vivo control of OX40 signals and thus normal functions of OX40 signals may be obscured. However, in the response to Lm-2W at least, we show that signals through OX40 are not required to generate a TFH-cell population. Furthermore, this population did not express OX40 in WT mice at a time when established GC structures were evident. Should TFH cells require OX40 signals for persistence, presumably these cells should express OX40, which may occur in situations of chronic infection and abundant antigen. This model would also require OX40L expression by a GC-resident cell type, which has not been clearly described. In the Lm-2W model, TFH cells are clearly reliant upon B-cell-derived ICOS ligand (ICOSL) for their generation 19. Therefore, amongst CD4^+^ T-cell subsets, distinct requirements for costimulatory molecules exist, which are also likely different for naive and memory cells.

Whilst there are considerable data supporting a role in the expansion and survival of CD4^+^ T cells during a primary response, direct evidence for memory CD4^+^ T-cell survival is lacking, particularly under physiological conditions. These studies were also prompted by efforts to understand the mechanisms by which ROR-γ^+^ ILCs might support memory CD4^+^ T-cell survival 10. Infection of ROR-γ^−/−^ mice with Lm-2W results in a normal primary response, but a significantly reduced memory cell pool, consistent with the theory that ROR-γ^+^ ILCs supported memory cells through provision of OX40L. To test this, we blocked OX40L signals in vivo and found that the survival of 2W1S-specific memory CD4^+^ T cells was not significantly affected. It remains possible that blockade of OX40L signals was incomplete or was not maintained for sufficient time to see significant changes in memory cell numbers. As we were unable to detect OX40 expression on these memory CD4^+^ T cells directly ex vivo, this would seem an unlikely mechanism through which survival signals are mediated.

Since agonistic anti-OX40 Abs are being used clinically, these data clarify that the cost of enhanced effector T-cell responses may be the loss of high-affinity Ab generated though the GC. Agonist Abs to OX40 have been used in a variety of tumour models with varying success and also in phase I clinical trials 2,27. Ligation of OX40 clearly dramatically enhances the number of effector CD4^+^ T cells, which may enhance tumour clearance. Stimulation through OX40 is also being considered as a means of enhancing vaccine responses 2. Our data suggest that, depending on the context of the response, this approach may be less successful if high-affinity Ab responses are desired. Furthermore, despite the dramatic enlargement of the effector T-cell pool, numbers of antigen-specific T cells at the memory phase of the response were comparable between treated and control mice, perhaps due to poor conversion to memory cells as observed when non-physiological numbers of TCR transgenic T cells are analysed 15. In summary, our data clarify the importance of OX40 signals for physiological responses and provide data on the likely effects of clinical manipulation of OX40 signals in patients.

## Materials and methods

### Mice

Mice used were C57BL/6 (obtained from Harlan) and Rag1-Cre Tbx21 fl/fl Rosa26-tdRFP (Tbx21 fl/fl mice were kind gift from Dr. Steve Reiner to Dr. Marc Veldhoen). CD30^−/−^OX40^−/−^ mice 8 were compared to C57Bl/6 mice bred in-house. Animals were used in accordance with Home Office guidelines at the University of Birmingham and Babraham Institute, Biomedical Services Units.

### Immunisation

Mice were immunised intravenously (i.v.) with 10^7^ ActA-deficient Lm-2W (kind gift from Dr. Marc Jenkins) as described 16. Bacteria were grown at 37°C in a shaking incubator to concentration OD_600_ = 0.1 in Luria-Bertani broth supplemented with 20 μg/mL chloramphenicol. To generate a Th2 response, mice were immunised intra-peritoneally (i.p.) with 100 μg aluminium hydroxide precipitated 2W1S peptide. For in vivo peptide challenge, mice were injected i.v. with 100 μg 2W1S peptide with 2.5 μg LPS, and secondary lymphoid tissues were harvested 4 hours later for flow cytometric analysis.

### Ab injection

Blocking mAbs against mouse OX40L (clone RM134L) were prepared as described previously 28. Control rat IgG was purchased from Sigma-Aldrich. Mice were injected i.p. with 0.25 mg anti-OX40L or control rat IgG. For memory cell experiments, mice were injected twice weekly for 4 weeks at 28 dpi. To assess effect of blocking OX40 on primary responses, mice were injected 1 and 3 dpi. Mice were injected i.p. with 100 μg anti-OX40 (clone OX86) on the day of challenge for memory experiments or as stated.

### Flow cytometry

For tetramer staining, cells from secondary lymphoid tissue were pooled and stained for 1 hour at room temperature with 10 nm PE-conjugated 2W1S:I-A^b^. For in vivo peptide experiments, 10 μg/mL brefeldin A was added at this stage. All cell surface staining was done at 4°C for 30 min, with the exception of CXCR5 that was stained for 1 hour at room temperature. Enrichment for 2W1S:I-A^b^-specific T cells was performed at d2, d3, d4 and d28, as described previously, using anti-PE MicroBeads (Miltenyi Biotech) and MACS enrichment 29. Enriched and run-through fractions were stained with the same cocktail of surface Abs to enable calculation of cell frequencies. Samples were run using an LSR11 (BD Biosciences) and analysed using FlowJo software (Tree Star). For intracellular cytoplasmic staining, cells were fixed and permeabilised with Cytofix/Cytoperm Plus (BD Biosciences) according to manufacturer's instructions. Intracellular cytokines were stained with IL-2 488 and IFN- γ PECy7 (BD Bioscience).

### Immunofluorescence and image analysis

Tissues sections from experimental mice were cut and stained as described previously 10. Sections were counterstained with DAPI (Invitrogen) and mounted using ProLong Gold (Invitrogen). Slides were analysed on an LSM 510 Meta confocal (Zeiss). For calculations of number and size of GCs, tile scans of whole spleen sections were made, and all Bcl-6^+^ cell clusters were identified and the area was calculated using LSM (Zeiss).

### Statistics

Data were analysed using GraphPad Prism (version 5.01). Non-parametric Mann–Whitney test was used to determine significance that was set at *p* ≤ 0.05. Median values were calculated and used in all analyses.

## Abbreviations

dpi: days post-infection
ILC: innate lymphoid cell
LCMV: lymphocytic choriomeningitis virus
Lm-2W: *Listeria monocytogenes* expressing 2W1S peptide
PD-1: programmed death-1
TFH: T follicular helper
