# Supplementary material for: OX40 controls effector CD4+ T-cell expansion, not follicular T helper cell generation in acute Listeria infection
Source: Eur J Immunol. 2014 May 21;44(8):2437–47. doi: 10.1002/eji.201344211 (PMC4285916; doi:10.1002/eji.201344211)
Supplement: Supplementary file 1 — Supplementary [file eji0044-2437-SD1.pdf]

# European Journal of Immunology

## Supporting Information
## for
## DOI 10.1002/eji.201344211


Clare L. Marriott, Emma C. Mackley, Cristina Ferreira, Marc Veldhoen, Hideo Yagita and David R. Withers

**OX40 controls effector CD4$^{+}$ T-cell expansion, not follicular T helper cell generation in acute *Listeria* infection**

© 2014 The Authors. *European Journal of Immunology* published by WILEY-VCH Verlag GmbH & Co. KGaA, Weinheim

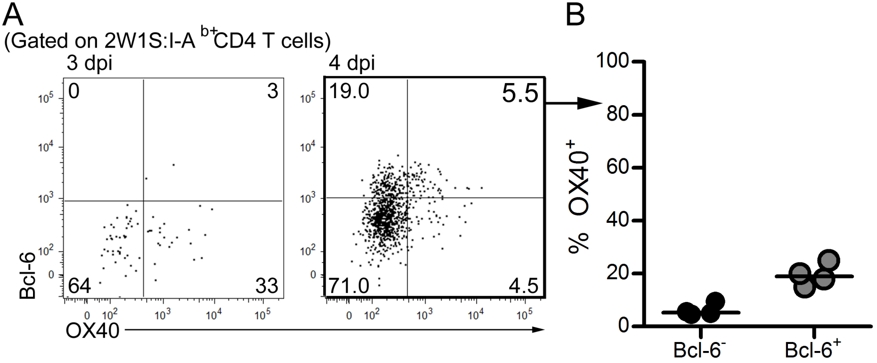
A
(Gated on 2W1S:I-A b+CD4 T cells)
3 dpi
4 dpi
0
3
64
33
19.0
5.5
71.0
4.5
Bcl-6
OX40
B
% OX40+
100
80
60
40
20
0
Bcl-6-
Bcl-6+

Figure S1

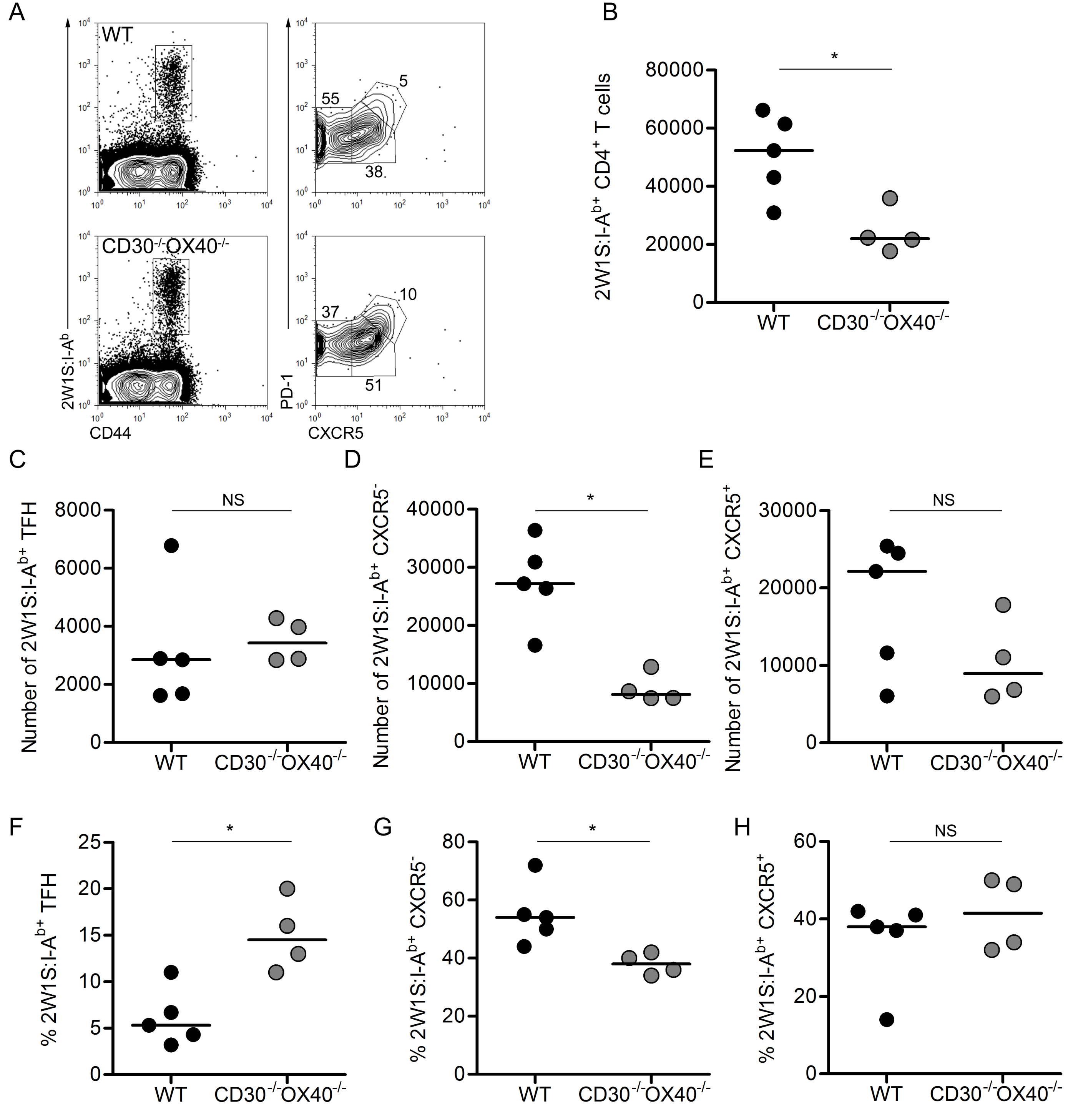
A
WT
CD30-/-OX40-/-
2W1S:I-Ab
CD44
PD-1
CXCR5
55
5
38
37
10
51
B
2W1S:I-Ab+ CD4+ T cells
WT
CD30-/-OX40-/-
C
Number of 2W1S:I-Ab+ TFH
NS
D
Number of 2W1S:I-Ab+ CXCR5-
*
E
Number of 2W1S:I-Ab+ CXCR5+
NS
F
% 2W1S:I-Ab+ TFH
*
G
% 2W1S:I-Ab+ CXCR5-
*
H
% 2W1S:I-Ab+ CXCR5+
NS

Figure S2

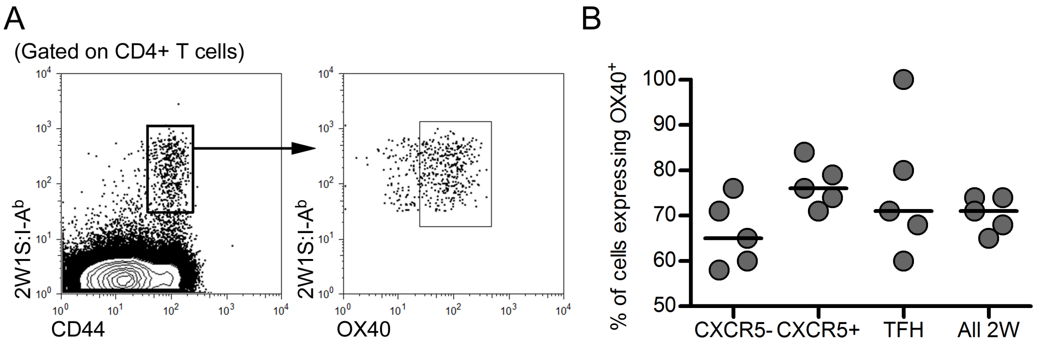
A
(Gated on CD4+ T cells)
2W1S:I-Ab
CD44
OX40
B
% of cells expressing OX40+
CXCR5-
CXCR5+
TFH
All 2W

Figure S3

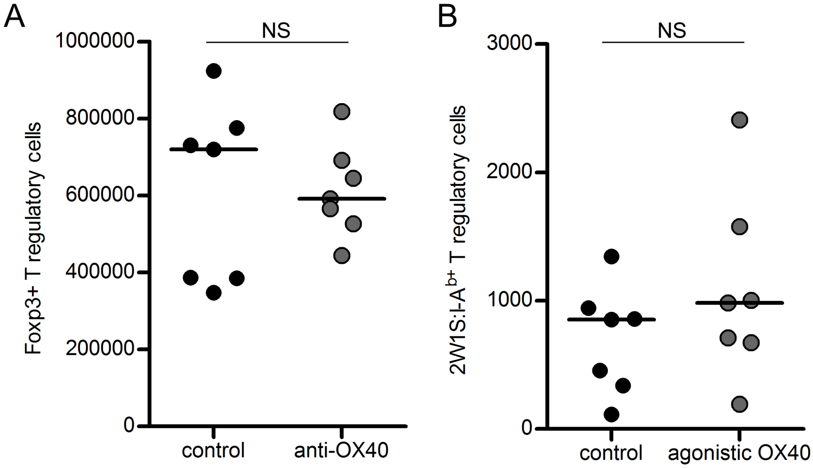
A
NS
1000000
800000
600000
400000
200000
0
Foxp3+ T regulatory cells
control
anti-OX40
B
NS
3000
2000
1000
0
2W1S:I-Ab+ T regulatory cells
control
agonistic OX40

Figure S4

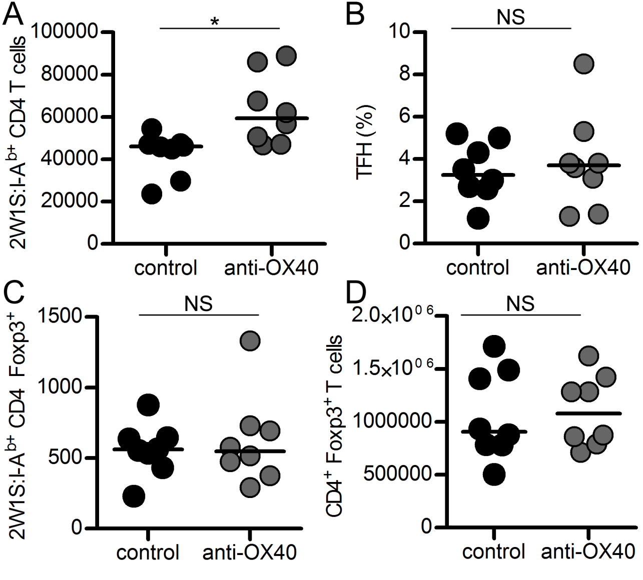
A
2W1S:I-Ab+ CD4 T cells
100000
80000
60000
40000
20000
0
*
control
anti-OX40
B
TFH (%)
10
8
6
4
2
0
NS
control
anti-OX40
C
2W1S:I-Ab+ CD4 Foxp3+
1500
1000
500
0
NS
control
anti-OX40
D
CD4+ Foxp3+ T cells
2.0x10 0 6
1.5x10 0 6
1000000
500000
0
NS
control
anti-OX40

Figure S5

**Supporting Information Figure 1. Bcl-6 is expressed on 2W1S:I-A$^{b+}$ CD4$^{+}$ T cells at 4 days post immunisation.** Analysis of Bcl-6 expression at 3 and 4 dpi with Lm-2W. (A) Detection of Bcl-6 on 2W1S:I-A$^{b+}$ CD4$^{+}$ T cells at 3 and 4 dpi. Gating strategy: plots are gated on 2W1S:I-A$^{b+}$ CD4$^{+}$ T cells. (B) Percentage of Bcl-6$^{-}$ and Bcl-6$^{+}$ cells expressing OX40 at 4 dpi. (A) Plots are representative of 4 mice from 2 independent experiments. (B) Graph shows pooled data from 2 independent experiments, each point represents 1 mouse. Bars show medians.

**Supporting Information Figure 2. OX40 is not required for the generation of 2W1S-specific T follicular helper cells.** Analysis of CD30$^{-/-}$OX40$^{-/-}$ mice 7 dpi with Lm-2W. (A) Representative CXCR5 and PD-1 staining on CD44$^{hi}$ 2W1S:I-A$^{b+}$ CD4$^{+}$ T cells in WT and CD30$^{-/-}$OX40$^{-/-}$. (B) Enumeration of CD44$^{hi}$ 2W1S:I-A$^{b+}$ CD4$^{+}$ T cells in the spleen. Total number of CD44$^{hi}$ 2W1S:I-A$^{b+}$ CD4$^{+}$ T cells that are (C) CXCR5$^{+}$ PD-1$^{+}$ TFH cells, (D) CXCR5$^{-}$ effector cells, or (E) CXCR5$^{+}$ cells. Percentage of CD44$^{hi}$ 2W1S:I-A$^{b+}$ CD4$^{+}$ T cells that are (F) CXCR5$^{+}$ PD-1$^{+}$ TFH cells, (G) CXCR5$^{-}$ effector cells, or (H) CXCR5$^{+}$ cells. (A) Plots are representative of ≥4 mice from 2 independent experiments. (B-H) Graphs show pooled data from 2 independent experiments, each point represents 1 mouse. Bars show medians. Mann-Whitney test * = $p<0.05$, NS = non-significant.

**Supporting Information Figure 3. OX40 is rapidly upregulated on CD44$^{hi}$ 2W1S:I-A$^{b+}$ CD4$^{+}$ T cells post stimulation with peptide.** Mice were immunised

with Lm-2W and restimulated on 7 dpi with 2W1S peptide; spleen was analysed 4 hours post stimulation. (A) OX40 expression on CD44$^{hi}$ 2W1S:I-A$^{b+}$ CD4$^{+}$ T cells. (B) Percentage of CXCR5$^{-}$, CXCR5$^{+}$ and TFH cells amongst CD44$^{hi}$ 2W1S:I-A$^{b+}$ CD4$^{+}$ T cells, or all of CD44$^{hi}$ 2W1S:I-A$^{b+}$ CD4$^{+}$ T cells, expressing OX40 4 hours post stimulation. (A) Plots are representative of 5 mice from 2 independent experiments. (B) Graph shows pooled data from 2 independent experiments, each point represents 1 mouse

**Supporting Information Figure 4. Foxp3$^{+}$ T regulatory cells are not depleted by agonist anti-OX40 Abs.** Total CD4$^{+}$ Fop3$^{+}$ Treg cells (A) or 2W1S:I-A$^{b+}$CD4$^{+}$Foxp3$^{+}$ Treg cells (B) 7 dpi in mice given control IgG or anti-OX40 Abs 1 dpi. Graphs show pooled data from 2 independent experiments, bars show medians. Mann-Whitney test NS = non-significant.

**Supporting Information Figure 5. OX40 ligation only affects effector cell expansion and phenotype during early stages of the primary response.** Analysis of CD44$^{hi}$ 2W1S:I-A$^{b+}$ CD4$^{+}$ T cells in spleen 7 dpi from mice given control IgG or anti-OX40 Abs 4 dpi. (A) Enumeration of CD44$^{hi}$ 2W1S:I-A$^{b+}$ CD4$^{+}$ T cells. (B) Percentage of CD44$^{hi}$ 2W1S:I-A$^{b+}$ CD4$^{+}$ T cells that are CXCR5$^{+}$ PD-1$^{+}$ TFH cells. (C) Enumeration of 2W1S:I-A$^{b+}$ CD4$^{+}$ Foxp3$^{+}$ Treg cells or (D) total CD4$^{+}$Foxp3$^{+}$ Treg cells. (A-D) Graphs show pooled data from 2

independent experiments, bars show medians. Mann-Whitney test * = $p<0.05$, NS = non-significant.
