# Supplementary material for: OX40 controls effector CD4+ T-cell expansion, not follicular T helper cell generation in acute Listeria infection
Source: Eur J Immunol. 2014 May 21;44(8):2437–47. doi: 10.1002/eji.201344211 (PMC4285916; doi:10.1002/eji.201344211)
Supplement: Supplementary file 2 — Supplementary [file eji0044-2437-SD2.pdf]

European Journal of Immunology

Peer review correspondence

OX40 controls effector CD4$^+$ T-cell expansion, not follicular T helper cell generation in acute *Listeria* infection

Clare L. Marriott, Emma C. Mackley, Cristina Ferreira, Marc Veldhoen, Hideo Yagita and David R. Withers.

Corresponding author: David R. Withers, MRC Centre for Immune Regulation, University of Birmingham

---

Review Timeline:	Submission date:	25 October 2013
	First editorial decision:	26 November 2013
	First revision received:	18 February 2014
	Second editorial decision:	14 March 2014
	Second revision received:	25 March 2014
	Accepted:	17 April 2014

Handling Executive Committee member: Prof. Shimon Sakaguchi

Please note that the correspondence below does not include the standard editorial instructions regarding preparation and submission of revised manuscripts, only the scientific revisions requested and addressed.

First Editorial Decision – 26 November 2013

Dear Dr. Withers,

Manuscript ID eji.201344211 entitled "Heterogenous OX40 expression underlies preferential role in effector cell expansion, not memory cell survival nor follicular T Helper cell generation." which you submitted to the European Journal of Immunology has been reviewed. The comments of the referees are included at the bottom of this letter.

You will see that referee 1 is unconvinced by the data in your study and furthermore feels that your study was in some ways scooped by the publication by Boettler et al. in the Journal of Immunology last month. You should know that this referee recommended that we reject your manuscript on these grounds; although we do not fully agree with this referee we do strongly encourage you to address this referee's concerns in your rebuttal or discussion, and to highlight the advance of your work beyond that of Boettler

European Journal of Immunology

et al. Therefore, a revised version of your manuscript that takes into account the comments of the referees will be reconsidered for publication.

You should also pay close attention to the editorial comments included below. *In particular, please edit your figure legends to follow Journal standards as outlined in the editorial comments. Failure to do this will result in delays in the re-review process.*

Please note that submitting a revision of your manuscript does not guarantee eventual acceptance, and that your revision will be re-reviewed by the referees before a decision is rendered.

If the revision of the paper is expected to take more than three months, please inform the editorial office. Revisions taking longer than six months may be assessed by new referees to ensure the relevance and timeliness of the data.

Once again, thank you for submitting your manuscript to European Journal of Immunology and we look forward to receiving your revision.

Yours sincerely,
Karen Chu

On behalf of Prof. Shimon Sakaguchi

Editorial Office
European Journal of Immunology
e-mail: ejied@wiley.com

****

Reviewer: 1

Comments to the Author
The authors suggest that the expression of OX40 on polyclonal T cells is different than on the previously studied transgenic T cells, however, it seems more likely that it isn't TCR transgenic versus polyclonal that is different but how the T cells are stimulated and the persistence of Ag. A study by Dawicki et al. The Journal of Immunology, 2004, 173: 5944–5951 also showed OX40 was detected on OT-II cells at 24 and 48hr but gone by 72hrs. Thus it is likely that OX40 expression reflects its transient induction by TCR signaling rather than transgenic versus polyclonal responses. There are transgenic models that can be used with the attenuated Listeria model, in which Listeria expresses ova for example, so to make these

European Journal of Immunology

claims that the results are different due to transgenic frequency and not due to different presentation of Ag, they need to compare Transgenic and polyclonal responses within the same study using the same Ag/delivery system… I suspect they would find similar results. As the present study uses a highly attenuated Listeria, it may be rapidly controlled, limiting TCR signaling. Indeed, this interpretation fits with the finding that OX40 is restricted to CD25+ (or activated) T cells. Others have shown that the related TNFR family member 4-1BB comes up transiently with CD69 and then is downregulated using a TCR transgenic model and a non-replicating antigen. Therefore, to make the case that studies with TCR transgenics give different results, the authors need to compare them in the same study with the same immunization scheme, as the rate of antigen presentation and the rate of antigen clearance likely dictate the duration of OX40 expression on the effector T cells.

The authors conclude in figure 1, that OX40 is not expressed on Tfh, because they only detect OX40 on the CD25+ population. However, there may be too few Tfh to detect at this time point. The figure showing CD25x OX40 has very few events and there is no staining of Tfh in this figure that I could see. The authors need to track Tfh with markers and determine on a reasonably sized population over time if OX40 is expressed. Does OX40 get upregulated on the Tfh cells upon restimulation- so if one uses peptide stimulation do the CXCR5x PD1hi x Bcl6+ T cells express OX40- as it is possible the kinetics of OX40 expression on the Tfh is just different and the times examined did not look at a time when Tfh are present in sufficient numbers and activated?

I don’t see how you can conclude on page 8 that OX40 is restricted to effector cells- using only CD25- I think it may be restricted to activated T cells, as measured by CD25- a comparison with CD69 would be of interest. It may be that the kinetics of response is not synchronous in the polyclonal T cells, so that at any one time only a fraction of the detected cells are OX40 positive.

The idea that OX40 agonist prevents Tfh activity by competition is supported by a decreased density of Tfh in the GC. However, it is possible that there are other explanations- do cytokines produced by the expanding effectors limit the Tfh response? Or prevent their entry in the GC?

The study of Boettler et al. published online Oct 7, comes to similar conclusions about OX40 limiting Tfh responses, using LCMV clone 13 and Armstong models and examined both polyclonal and transgenic responses. Thus the authors need to clarify their argument that the role of OX40 has not been studied on polyclonal T cells. The paper of Boettler et al. used non-transgenic B6 mice treated with anti-OX40 agonist to show impaired GC response. In this case they show that it is CD8 T cells that are disrupting the GC reaction, which is perhaps distinct from this model, as in the LCMV clone 13 model, CD8 T cells cause pathology.

Indeed, other studies, notably by Van Lier using a CD70 transgenic model or studies from the Ochsenbein lab (Matter et al. 20060 have also shown that strong costimulation through CD27 causes pathology and loss of B cell responses. In the Van Lier study (Arens et al. Immunity 2001), chronic costimulation through CD27 led to IFNgamma mediated B cell depletion.
Thus one wonders if the Tfh defects in this model –such as low frequency in the spleen could be due to indirect effects of anti-OX40 inducing cytokines that disrupt the recruitment of these cells via affects on B cells or the organization of the GC. Thus I find based on the data provided the study is quite over-interpreted as evidence of competition. Whether the effects are due to there being more T cells or due to their cytokines affecting bystander cells is not clear from the present study.

The abstract indicates that OX40 is not required for Tfh generation, but their data are insufficient to conclude this definitively. The evidence that Tfh do not express OX40 during this response is rather weak. Moreover, they are using a bacterial model in which cell mediated rather than antibody responses are more important in pathogen control, so perhaps the wrong model to test this. To definitively rule out a role for OX40 in the Tfh generation one would like to see use of k/o of receptor or ligand. The absence of detection of the receptor or the finding that a supraphysiological signal through OX40 decreases the response is not convincing as to the dispensability of OX40 for the Tfh response.

Reviewer: 2

Comments to the Author

The paper reports results manipulating OX40 with blocking or agonist antibodies in response to attenuated Listeria. Overall the results are not tremendously new in terms of understanding the activity of OX40 and its ligand. However, they are still a reasonable addition to the literature as it is important to understand that OX40 expression can be restricted depending on the infection or immunization context, and that ligating OX40 can result in select outgrowth of subpopulations of T cells.

The addition of some minor analyses would make the paper a little more easier to interpret.

1. Fig. 1. The authors conclude that OX40 is only selectively expressed on a subset of CD4 T cells with attenuated listeria infection. It is obvious that few T cells express OX40 on day 4 after infection and onwards, but expression of OX40 particularly in relation to others markers is very difficult to evaluate early in the response due to the low numbers of antigen-reactive T cells. The authors suggest that only a proportion of effector and not Tfh or pre-Tfh cells express OX40 based on CD25 staining but they refer to cells that can be CXCR5 or PD-1+/- to differentiate between Tfh and Th1 without showing costaining for these molecules. From the data presented, it is difficult to know if their conclusions are correct as so few

cells are analyzed. The authors use the system developed by Jenkins for immunization, and so they should also enrich for the tetramer+ cells as done by the same lab and pool cells from multiple mice to allow a reasonable evaluation of the expression of OX40 on different T cell subsets (effector vs Tfh, etc) on days 2 and 3.

2. Fig. 2. The authors inject an agonist to OX40 at the time of secondary challenge and show the number of Tfh are the same, but it is not clear whether the GC response is or is not affected. The data on GC are represented per 1000 um2 area. To have a clear picture, given the reported increase in spleen size, the authors should show the total number of GC per spleen, which may be similar to control mice given that the Tfh numbers were not changed.

3. Fig. 6. The total numbers of Tfh vs effectors should also be shown to allow comparison to the prior data with listeria. Also, with alum immunization, are the total number of GC reduced after anti-OX40 treatment.

4. The authors make a statement about signals other than OX40 controlling the generation of Tfh in the discussion (page 16). While there are data that say OX40 signaling may be irrelevant for Tfh development, other data show that it is very important (ref 21, Boettler with chronic LCMV) and refs from Lane et al where OX40 and CD30 are both inactive. Therefore the requirement of Tfh for OX40 is likely context dependent as is the importance to effector memory and central memory T cell development.

Minor: Fig. 6B. Graphs not labeled for anti-OX40

First revision – authors' response – 18 February 2014

We thank the reviewers for their comments and have addressed each point individually in red italics below. Changes to the text are shown in red and the page number is given. The summary and discussion are completely re-written. The title has been amended to better reflect our findings.

Reviewer: 1

Comments to the Author

1. The authors suggest that the expression of OX40 on polyclonal T cells is different than on the previously studied transgenic T cells, however, it seems more likely that it isn't TCR transgenic versus polyclonal that is different but how the T cells are stimulated and the persistence of Ag. A study by Dawicki et al. The Journal of Immunology, 2004, 173: 5944–5951 also showed OX40 was detected on OT-II cells at 24 and 48hr but gone by 72hrs. Thus it is likely that OX40 expression reflects its transient induction by TCR signaling rather than transgenic versus polyclonal responses. There are transgenic models that can be used with the attenuated Listeria model, in which Listeria expresses ova for example, so to make these claims that the results are different due to transgenic frequency and not due to different presentation of Ag, they need to compare Transgenic and polyclonal responses within the same study

European Journal of Immunology

using the same Ag/delivery system… I suspect they would find similar results. As the present study uses a highly attenuated Listeria, it may be rapidly controlled, limiting TCR signaling. Indeed, this interpretation fits with the finding that OX40 is restricted to CD25+ (or activated) T cells.   Others have shown that the related TNFR family member 4-1BB comes up transiently with CD69 and then is downregulated using a TCR transgenic model and a non-replicating antigen. Therefore, to make the case that studies with TCR transgenics give different results, the authors need to compare them in the same study with the same immunization scheme, as the rate of antigen presentation and the rate of antigen clearance likely dictate the duration of OX40 expression on the effector T cells.

We agree with the reviewer that the type of antigen and the nature of the exposure are important in determining the kinetics of OX40 expression. We sought to make the point that existing studies have only provided expression data for OX40 using monoclonal TCR transgenic T cells, whilst physiological responses consist of a polyclonal response and in this context, OX40 expression may not by uniformly expressed. We have amended our descriptions to simply state that we sought to assess a physiological CD4 T cell response. We thought it of interest that in a polyclonal primary response OX40 was not expressed by all responding cells, in contrast to what has been published previously using monoclonal TCR transgenic T cells.

In the discussion we have tried to make clear that the nature of the antigen and the adjuvant clearly affect the kinetics of OX40 expression and put our data in context with other studies.

We performed the experiment described by the reviewer by transferring OTII cells into WT mice and immunising with Lm-2W. We further investigated whether a starting population of 10,000 or 1,000,000 OTII cells affected the kinetics of OX40 expression. Surprisingly, in either situation, we could detect very little OX40 expression at early time points (2,3,4 and 7 dpi) despite good T cell expansion indicating different kinetics of OX40 expression on TCR transgenic cells immunised with the same antigen. Since these experiments seemed to distract from the focus of our manuscript, understanding a physiological CD4 T cell response, we have not included this data.

2. The authors conclude in figure 1, that OX40 is not expressed on Tfh, because they only detect OX40 on the CD25+ population. However, there may be too few Tfh to detect at this time point. The figure showing CD25x OX40 has very few events and there is no staining of Tfh in this figure that I could see. The authors need to track Tfh with markers and determine on a reasonably sized population over time if OX40 is expressed. Does OX40 get upregulated on the Tfh cells upon restimulation- so if one uses peptide stimulation do the CXCR5x PD1hi x Bcl6+ T cells express OX40- as it is possible the kinetics of OX40 expression on the Tfh is just different and the times examined did not look at a time when Tfh are present in sufficient numbers and activated?

European Journal of Immunology

We agree with the reviewer that the original data did not make it clear and as suggested by Reviewer 2 we have pooled samples from several mice to increase the number of 2W-specific cells analysed. We have provided additional analysis (Figure 2E, F and G) showing the majority of cells express CD25, which others have used as an effector cell marker (e.g. Pepper at al., 2011; Boettler et al., 2013). This data and description is present on Page 7.

We agree with the reviewer that our conclusions concerning TFH required direct assessment of TFH markers. We were unable to detect expression of either Bcl-6 or CXCR5 at 3dpi, when most of the cells expressed OX40. We could detect Bcl-6 at 4dpi, when some 2W1S-specific CD4 T cells retained OX40 expression. Analysis at this time point revealed a small population of Bcl-6+ OX40+ cells. We would therefore agree that it is not possible to state that no TFH cells express OX40 and have revised our description of the data and included the additional Bcl-6 staining as a new Supplementary Figure 1. We would make the point that the majority (75%+) of the cells expressing OX40 express CD25, which has been published in this model as an effector cell marker. This data and description is present on Page 6.

As proposed by the reviewer, we challenged mice at 7dpi with 2W1S peptide and observed that the majority of 2W1S-specific CD4 T cells rapidly express OX40 as witnessed at the later 'memory' timepoint. This data is included as Supplementary Figure 3 and we have tried to make clear that this provides further evidence of antigen availability dictating OX40 expression.
This data and description is present on Page 7.

We would make the point that we cannot detect any OX40 expression on TFH cells directly ex vivo when established GCs are present. Whilst we cannot exclude that TFH cells express OX40 at time points other than those analysed, our data indicate that the cells do not constitutively express this marker. We have amended our description of the data to make this clear. Also, further experiments requested by the reviewer (point 6.) indicate that OX40 is not required for TFH generation in this response.

Finally, in our discussion we try to make clear that TFH requirements may differ depending upon the nature of the infection/response and we have sought to better put our data in context with other studies.

3. I don't see how you can conclude on page 8 that OX40 is restricted to effector cells- using only CD25- I think it may be restricted to activated T cells, as measured by CD25- a comparison with CD69 would be of interest. It may be that the kinetics of response is not synchronous in the polyclonal T cells, so that at any one time only a fraction of the detected cells are OX40 positive.

We used CD25 expression as a marker of effector cells as published by the group who first described the response to Lm-2W (Pepper at al. 2011). We note that others (e.g. Boettler at al. 2013) have also used CD25 to identify effector cells. The number of 2W1S-specific CD25- cells detected is significantly more

European Journal of Immunology

than the total naïve 2W1S-specific population, indicating activation and expansion of this subset. An example of the number of naïve 2W1S-specific CD4 T cells is shown below:

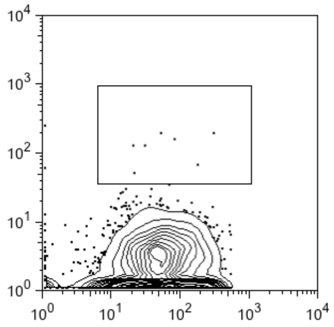

As suggested by the reviewer, we did look at CD69 expression at 3dpi, the time point when OX40 was detectable. We could detect some CD69 expression on CD25+ and CD25- cells. This data is shown below for reference (two representative plots gated on CD4+ 2W1S-specific CD4 T cells). Also CD69 expression was not detected on all OX40+ cells. We did not feel this data added to the manuscript and thus have not included it.

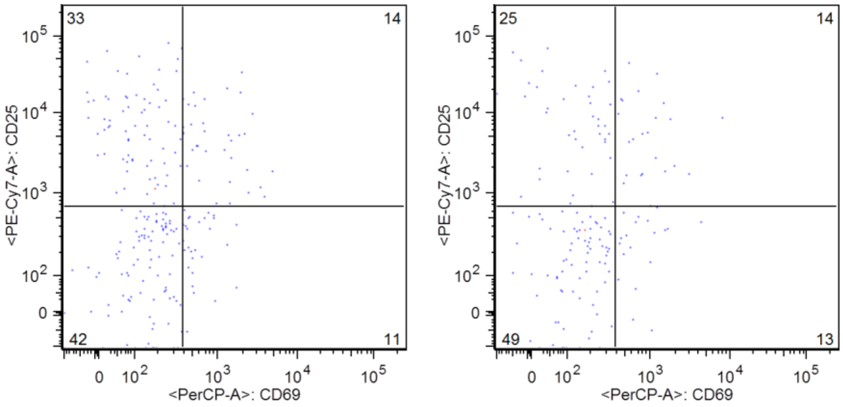

In our gating strategy we use CD44 expression to identify activated 2W1S+ cells. We provide data on 2, 3, 4, and 7 dpi in the primary response since these time points covered when we found OX40 expression to be off, switched on and then turned off again. We find that amongst the CD44hi cells, OX40 expression is briefly expressed and is then not detected unless antigen is re-encountered (e.g. in the form of injected 2W1S peptide).

4. The idea that OX40 agonist prevents Tfh activity by competition is supported by a decreased density of Tfh in the GC. However, it is possible that there are other explanations- do cytokines produced by the expanding effectors limit the Tfh response? Or prevent their entry in the GC?

European Journal of Immunology

We agree with the reviewer that cytokine production is likely important and with further consideration of the Boettler data (and other manuscripts such as Pepper et al. 2011), enhanced IL-2 production is probably the key mechanism driving the enhanced effector population. It remains unclear why in the primary response the TFH population is essentially lost. We have revised our description and discussion of this data and stated that in the primary response there may be out-competition.

5. The study of Boettler et al. published online Oct 7, comes to similar conclusions about OX40 limiting Tfh responses, using LCMV clone 13 and Armstong models and examined both polyclonal and transgenic responses. Thus the authors need to clarify their argument that the role of OX40 has not been studied on polyclonal T cells. The paper of Boettler et al. used non-transgenic B6 mice treated with anti-OX40 agonist to show impaired GC response. In this case they show that it is CD8 T cells that are disrupting the GC reaction, which is perhaps distinct from this model, as in the LCMV clone 13 model, CD8 T cells cause pathology.

The Study of Boettler et al. does show data describing the effects of anti-OX40 antibodies on a polyclonal response tracking endogenous viral specific CD4 T cells (recognising GP66) using MHCII tetramers. They show that for this population, there is skewing towards the effector subset after OX40-ligation, consistent with their Smarta TCR transgenic T cell data. We were trying to make the point that amongst the studies by Boettler at al in 2012 and 2013, the only OX40 expression data we could find for CD4 T cells responding to LCMV came from Smarta TCR transgenic T cells, which all expressed OX40 at 3dpi.

Whilst the mechanism proposed by Boettler et al. may be occurring in our model, since most of the cells that express OX40 are effector cells, we considered that expansion of this population does not require conversion from another developing subset. We would agree that there was too little discussion of our data in comparison with that of Boettler et al., and have addressed this in the discussion.

Indeed, other studies, notably by Van Lier using a CD70 transgenic model or studies from the Ochsenbein lab (Matter et al. 20060 have also shown that strong costimulation through CD27 causes pathology and loss of B cell responses. In the Van Lier study (Arens et al. Immunity 2001), chronic costimulation through CD27 led to IFNgamma mediated B cell depletion.
Thus one wonders if the Tfh defects in this model –such as low frequency in the spleen could be due to indirect effects of anti-OX40 inducing cytokines that disrupt the recruitment of these cells via affects on B cells or the organization of the GC. Thus I find based on the data provided the study is quite over-interpreted as evidence of competition. Whether the effects are due to there being more T cells or due to their cytokines affecting bystander cells is not clear from the present study.

6. The abstract indicates that OX40 is not required for Tfh generation, but their data are insufficient to conclude this definitively. The evidence that Tfh do not express OX40 during this response is rather

European Journal of Immunology

weak. Moreover, they are using a bacterial model in which cell mediated rather than antibody responses are more important in pathogen control, so perhaps the wrong model to test this. To definitively rule out a role for OX40 in the Tfh generation one would like to see use of k/o of receptor or ligand. The absence of detection of the receptor or the finding that a supraphysiological signal through OX40 decreases the response is not convincing as to the dispensability of OX40 for the Tfh response.

We agree with the reviewer's comment and to better address this we have immunised OX40-/-CD30-/- mice with Lm-2W and looked at the TFH cell population at 7dpi. We find normal numbers of the TFH cells at this timepoint but much reduced numbers of effector CD4 T cells indicating that this population, rather than TFH cells require OX40 for their generation. This data is present as a new Supplementary Figure 3 and described on Page 7. We agree that this may reflect a response to an acute bacterial infection and in other contexts, TFH cells may be influenced by OX40 expression. We have amended the discussion to take this into account and we have also changed the title to reflect this.

Reviewer: 2

Comments to the Author

The paper reports results manipulating OX40 with blocking or agonist antibodies in response to attenuated Listeria. Overall the results are not tremendously new in terms of understanding the activity of OX40 and its ligand. However, they are still a reasonable addition to the literature as it is important to understand that OX40 expression can be restricted depending on the infection or immunization context, and that ligating OX40 can result in select outgrowth of subpopulations of T cells.

The addition of some minor analyses would make the paper a little more easier to interpret.

1. Fig. 1. The authors conclude that OX40 is only selectively expressed on a subset of CD4 T cells with attenuated listeria infection. It is obvious that few T cells express OX40 on day 4 after infection and onwards, but expression of OX40 particularly in relation to others markers is very difficult to evaluate early in the response due to the low numbers of antigen-reactive T cells. The authors suggest that only a proportion of effector and not Tfh or pre-Tfh cells express OX40 based on CD25 staining but they refer to cells that can be CXCR5 or PD-1+/- to differentiate between Tfh and Th1 without showing costaining for these molecules. From the data presented, it is difficult to know if their conclusions are correct as so few cells are analyzed. The authors use the system developed by Jenkins for immunization, and so they should also enrich for the tetramer+ cells as done by the same lab and pool cells from multiple mice to allow a reasonable evaluation of the expression of OX40 on different T cell subsets (effector vs Tfh, etc) on days 2 and 3.

European Journal of Immunology

We agree with the reviewer and performed further experiments as suggested, pooling cells from multiple mice and looked at further markers associated with Tfh. The pooled samples contained a clearer 2W1S-specific population and enabled a better analysis of expression of OX40 and CD25. This data is present within a revised Figure 1 (parts E,F,G) and described on Page 6.The TFH markers CXCR5 and PD-1 are not detectable at day 3 when most 2W-specific cells are OX40+. We stained for Bcl-6, but could only detect this from 4dpi, when most of the OX40 expression is lost. We have included this analysis as a revised Supplemental Figure 1 and on Page 6 we describe a small Bcl-6+ OX40+ population. We therefore cannot conclude that all TFH cells lack expression of OX40. As suggested by Reviewer 1, we analysed the response to Lm-2W in mice deficient in OX40 and CD30 and found normal TFH numbers, but a much reduced effector T cell population. We have amended our description of the data to reflect this, indicating htat in this response, whilst OX40 may be expressed by a minority of TFH cells, it is not required for their generation.

2. Fig. 2. The authors inject an agonist to OX40 at the time of secondary challenge and show the number of Tfh are the same, but it is not clear whether the GC response is or is not affected. The data on GC are represented per 1000 um2 area. To have a clear picture, given the reported increase in spleen size, the authors should show the total number of GC per spleen, which may be similar to control mice given that the Tfh numbers were not changed.

We take the reviewers point that our description of the data was not clear and have included data on the number of GCs per area of spleen tissue (Figure 2I).Although the absolute numbers of TFH cells are not reduced we do find fewer GCs per unit area of spleen We have attributed this effect to a decreased density of TFH cells due to increased spleen size and effector cell numbers in mice receiving agonist treatment.
This data and description is present on Page 9.

3. Fig. 6. The total numbers of Tfh vs effectors should also be shown to allow comparison to the prior data with listeria. Also, with alum immunization, are the total number of GC reduced after anti-OX40 treatment.

We have included this data in a revised Figure 6 and amended the text on Page 13 to describe this data. Total number of CXCR5- effector cells are significantly increased in mice receiving anti-OX40 in response to alum precipitated 2W1S peptide. Total number of TFH cells, however, are not significantly different despite a decreased percentage. These data are consistent with anti-OX40 driving effector cell expansion. The differences already discussed in terms of different responses affecting OX40 expression and kinetics may account for this. Further experiments beyond the scope of this manuscript could better explore OX40-ligation and the GC response after the use of alum adjuvant.

European Journal of Immunology

4. The authors make a statement about signals other than OX40 controlling the generation of Tfh in the discussion (page 16). While there are data that say OX40 signaling may be irrelevant for Tfh development, other data show that it is very important (ref 21, Boettler with chronic LCMV) and refs from Lane et al where OX40 and CD30 are both inactive. Therefore the requirement of Tfh for OX40 is likely context dependent as is the importance to effector memory and central memory T cell development.

We have amended our discussion to try and take into account antigen context and make clear that in different responses, TFH requirements for OX40 may differ. Using OX40-/-CD30-/- mice we have now demonstrated that OX40 expression is not required for the generation of TFH in response to Lm-2W. We sought to make the point that in at least some cases, OX40 expression was not required for the formation of TFH cells, a point that we thought was not clear in the literature. We would make the point that in the Lane lab studies, antigen specific populations of T cells were not really assessed and it is not clear why the GCs ‘breakdown’ in OX40-/-CD30-/- mice. The proposed model of TFH cells requiring OX40 survival signals may be true in some contexts, but this would require expression of OX40 when GC are present, which we do not see in the Lm-2W response. Within the revised discussion we have tried to better put our data into context with other studies. We have also amended the title of the manuscript to better reflect what our data shows, i.e. the context of the response.

Minor: Fig. 6B. Graphs not labeled for anti-OX40
We have amended our figure

Second Editorial Decision – 14 March 2014

Dear Dr. Withers,

Thank you for submitting your revised manuscript ID eji.201344211.R1 entitled "OX40 controls effector CD4 T cell expansion, not memory cell survival nor follicular T Helper cell generation in acute infection" to the European Journal of Immunology. Your manuscript has been re-reviewed and the comments of the referees are included at the bottom of this letter.

Although the referees have recommended publication, some revisions to your manuscript have been requested. Therefore, I invite you to respond to the comments of the referees and revise your manuscript accordingly.

If the revision of the paper is expected to take more than three months, please inform the editorial office. Revisions taking longer than six months may be assessed by new referee(s) to ensure the relevance and timeliness of the data.

Once again, thank you for submitting your manuscript to European Journal of Immunology. We look forward to receiving your revision.

Yours sincerely,
Laura Soto Vazquez

on behalf of Prof. Shimon Sakaguchi

Editorial Office
European Journal of Immunology
e-mail: ejied@wiley.com
www.eji-journal.eu

****

Reviewer: 1

Comments to the Author
The paper is much clearer and more convincing and the authors have clarified how the paper complements the recent study on OX40 in the LCMV model.

However, I still have a few minor issues:

The title of the paper is that OX40 controls effector CD4 T cell expansion but not memory cell survival nor follicular T helper generation in acute infection overstated with respect to memory T cells as the blocking experiment was not definitive. Thus I would leave the memory aspect out of the title.

The evidence that OX40 is dispensable for memory T cell survival is that adding antibody to OX40L twice a week between week 4 and 8 post infection does not significantly reduce the memory pool. However, a major caveat to this experiment is that we do not know that OX40L blockade is complete over this time period . If one compares the acute blocking of memory T cells in figure 1 with the blocking of effector response in figure 3, they actually show the same trend, the 2 fold effect on effectors is signficiant, but the slightly less than 2 fold at the memory stage- which is analyzed over a longer period, does not reach statistical significance, likely due to noise of the data. Thus I do not find this negative result particularly convincing as a stand alone and do not think this is sufficient evidence to put this as a major conclusion in the title/abstract unless its backed up with further data. Rather it would be better for the authors to focus on the positive result- and not make a strong conclusion on the memory point here.

European Journal of Immunology

The authors have introduced an OX40 x CD30 double knockout mouse strain to further support the lack of role for OX40 in Tfh generation. However, the source of mice are not mentioned in the methods. It appears that the mice are compared to purchased controls from Harlan. It is important to use littermates for this kind of experiment, as it is possible that changes in the two lines of mice, such as different flora, different numbers of regulatory (Tfr), incomplete backcrossing, could compensate for Tfh deficiency. As these data are used as a backup to the OX40 ligation studies, I think one could let this go, but the authors should clearly describe the knockout mice and their genetic background and the source of knockout and control mice. If these studies are to be used to refute the role of OX40 in Tfh, one would prefer a better controlled experiment- with littermate controls (or if the mice are highly backcrossed, at least cohoused controls to take care of flora issues). If the control mice were not completely appropriate, then the authors should acknowledge the limitation of these controls as a potential caveat in the discussion.

As discussed in my first review, I think that the expansion of the Teffector cells is likely to cause loss of Tfh in the primary response due to high levels of inflammatory cytokines. In a secondary response, where the response is more rapidly controlled or with peptide immunization, this may not be the case as observed by the authors. In the discussion the authors state that it suggests competition for niche by Tfh and Teffector but they do not acknowledge the possibility of toxic levels of IFN using supraphysiological OX40 signaling- this is conceptually similar to CD70 transgenic mice which cause B cell depletion and disruption of GC reaction due to too much IFN (see Arens et al. Immunity 15, 801). This possibility should be acknowledged in the discussion, as it may reflect a nonphysiological effect of OX40 rather than the normal role.

I think the conclusions about the dispensability of OX40L for memory are overstated and weaken the paper. This paragraph would be better removed from the paper and focus on the effect on Tfh and Teffector.

Reviewer: 2

Comments to the Author
The paper is much improved, particularly the discussion, which was re-written based on the concerns from both reviewers, and now gives a much more realistic and balanced view of how the current data integrate with other data on OX40. The new results that were added also adequately address most of the previous issues.

The primary comment relates to the title. Both reviewers remarked on the context in which OX40 has been studied, and the data already in the literature, which support the notion that the expression and contribution of OX40 varies depending on immunization, antigen, or infection. Given this, it seems

appropriate that the title of the paper should be more specific rather than using the term acute infection. For example, an acute infection with a replication-defective bacteria is very different from an acute infection with a virus. The authors should amend the title to "…generation in acute bacterial infection" or simply "…generation in acute Listeria infection"

Second revision – authors' response – 25 March 2014

Reviewer: 1

Comments to the Author
The paper is much clearer and more convincing and the authors have clarified how the paper complements the recent study on OX40 in the LCMV model.

However, I still have a few minor issues:

1. The title of the paper is that OX40 controls effector CD4 T cell expansion but not memory cell survival nor follicular T helper generation in acute infection overstated with respect to memory T cells as the blocking experiment was not definitive. Thus I would leave the memory aspect out of the title.

We have changed the title to:
'OX40 controls effector CD4+ T-cell expansion, not follicular T helper cell generation in acute Listeria infection'

2. The evidence that OX40 is dispensable for memory T cell survival is that adding antibody to OX40L twice a week between week 4 and 8 post infection does not significantly reduce the memory pool. However, a major caveat to this experiment is that we do not know that OX40L blockade is complete over this time period . If one compares the acute blocking of memory T cells in figure 1 with the blocking of effector response in figure 3, they actually show the same trend, the 2 fold effect on effectors is signficiant, but the slightly less than 2 fold at the memory stage- which is analyzed over a longer period, does not reach statistical significance, likely due to noise of the data. Thus I do not find this negative result particularly convincing as a stand alone and do not think this is sufficient evidence to put this as a major conclusion in the title/abstract unless its backed up with further data. Rather it would be better for the authors to focus on the positive result- and not make a strong conclusion on the memory point here.

We agree that the blocking OX40L experiments are not conclusive and have amended our discussion of the data. We do think that the lack of detectable OX40 expression (directly ex vivo) is important and

European Journal of Immunology

argues against constitutive expression of OX40 by memory cells as part of a survival mechanism. The discussion (Page 18) now reads:

‘To test this we blocked OX40L signals in vivo and found that the survival of 2W1S-specific memory CD4+ T-cells was not significantly affected. It remains possible that blockade of OX40L signals was incomplete or that the blockade was not maintained for sufficient time to see significant changes in memory cell numbers. As we were unable to detect OX40 expression on these memory CD4 T cells directly ex vivo, this would seem an unlikely mechanism through which survival signals are mediated.’

We have amended the abstract to remove mention of the blocking OX40L experiment. We wanted to make the point that memory 2W1S-specific CD4 T cells do not have detectable OX40 expression. The sentence in the abstract now reads:

‘OX40 was not expressed by 2W1S-specific memory cells, although it was rapidly upregulated upon challenge where upon Ab-ligation of OX40 specifically affected the effector subset.’

3. The authors have introduced an OX40 x CD30 double knockout mouse strain to further support the lack of role for OX40 in Tfh generation. However, the source of mice are not mentioned in the methods. It appears that the mice are compared to purchased controls from Harlan. It is important to use littermates for this kind of experiment, as it is possible that changes in the two lines of mice, such as different flora, different numbers of regulatory (Tfr), incomplete backcrossing, could compensate for Tfh deficiency. As these data are used as a backup to the OX40 ligation studies, I think one could let this go, but the authors should clearly describe the knockout mice and their genetic background and the source of knockout and control mice. If these studies are to be used to refute the role of OX40 in Tfh, one would prefer a better controlled experiment- with littermate controls (or if the mice are highly backcrossed, at least cohoused controls to take care of flora issues). If the control mice were not completely appropriate, then the authors should acknowledge the limitation of these controls as a potential caveat in the discussion.

For these experiments we compared the CD30-/-OX40-/- mice to WT mice bred in our animal facility. We have amended our description of this data in the Materials and Methods section on Page 19 to clarify this point:

‘CD30-/- OX40-/- mice [8] were compared to C57Bl/6 mice bred in house.’

4. As discussed in my first review, I think that the expansion of the Teffector cells is likely to cause loss of Tfh in the primary response due to high levels of inflammatory cytokines. In a secondary response, where the response is more rapidly controlled or with peptide immunization, this may not be the case as observed by the authors. In the discussion the authors state that it suggests competition for niche by Tfh

European Journal of Immunology

and Teffector but they do not acknowledge the possibility of toxic levels of IFN using supraphysiological OX40 signaling- this is conceptually similar to CD70 transgenic mice which cause B cell depletion and disruption of GC reaction due to too much IFN (see Arens et al. Immunity 15, 801). This possibility should be acknowledged in the discussion, as it may reflect a nonphysiological effect of OX40 rather than the normal role.

We agree with this point and have inserted the following sentence on Page 16

'Alternatively, the high levels of IFN produced following Ab-ligation of OX40 may cause the loss of TFH cells and GCs, analogous to CD70 transgenic mice {Arens, 2001 #1324}.'

I think the conclusions about the dispensability of OX40L for memory are overstated and weaken the paper. This paragraph would be better removed from the paper and focus on the effect on Tfh and Teffector.

Reviewer: 2

Comments to the Author

1. The paper is much improved, particularly the discussion, which was re-written based on the concerns from both reviewers, and now gives a much more realistic and balanced view of how the current data integrate with other data on OX40. The new results that were added also adequately address most of the previous issues.

The primary comment relates to the title. Both reviewers remarked on the context in which OX40 has been studied, and the data already in the literature, which support the notion that the expression and contribution of OX40 varies depending on immunization, antigen, or infection. Given this, it seems appropriate that the title of the paper should be more specific rather than using the term acute infection. For example, an acute infection with a replication-defective bacteria is very different from an acute infection with a virus. The authors should amend the title to "…generation in acute bacterial infection" or simply "…generation in acute Listeria infection"

We have changed the title to:

'OX40 controls effector CD4+ T-cell expansion, not follicular T helper cell generation in acute Listeria infection'

Peer review correspondence

European Journal of Immunology

Third Editorial Decision – 14 April 2014

Dear Dr. Withers,

It is a pleasure to provisionally accept your manuscript entitled "OX40 controls effector CD4+ T-cell expansion, not follicular T helper cell generation in acute Listeria infection" for publication in the European Journal of Immunology. For final acceptance, please follow the instructions below and return the requested items as soon as possible as we cannot process your manuscript further until all items listed below are dealt with.

Please note that EJI articles are now published online a few days after final acceptance (see Accepted Articles: http://onlinelibrary.wiley.com/journal/10.1002/(ISSN)1521-4141/accepted). The files used for the Accepted Articles are the final files and information supplied by you in Manuscript Central. You should therefore check that all the information (including author names) is correct as changes will NOT be permitted until the proofs stage.

We look forward to hearing from you and thank you for submitting your manuscript to the European Journal of Immunology.

Yours sincerely,
Karen Chu

on behalf of Prof. Shimon Sakaguchi

Dr. Karen Chu
Editorial Office
European Journal of Immunology
e-mail: ejied@wiley.com
www.eji-journal.eu
